# Human menstrual blood-derived stem cells reverse sorafenib resistance in hepatocellular carcinoma cells through the hyperactivation of mitophagy

**DOI:** 10.1186/s13287-023-03278-8

**Published:** 2023-04-01

**Authors:** Sining Zhou, Yiming Liu, Qi Zhang, Huikang Xu, Yangxin Fang, Xin Chen, Jiamin Fu, Yin Yuan, Yifei Li, Li Yuan, Charlie Xiang

**Affiliations:** 1 State Key Laboratory for Diagnosis and Treatment of Infectious Diseases, National Clinical Research Center for Infectious Diseases, National Medical Center for Infectious Diseases, Collaborative Innovation Center for Diagnosis and Treatment of Infectious Diseases, The First Affiliated Hospital, Zhejiang University School of Medicine, Hangzhou, 310003 China; 2 Laboratory of Cancer Biology, Key Laboratory of Biotherapy of Zhejiang Province, Sir Run Run Shaw Hospital, Zhejiang University School of Medicine, Hangzhou, 310016 China; 3 Department of Haematology, Affiliated Hangzhou First People’s Hospital, Zhejiang University, School of Medicine, Hangzhou, 310027 China; 4 Innovative Precision Medicine (IPM) Group, Hangzhou, 311215 China; 5 Research Units of Infectious Disease and Microecology, Chinese Academy of Medical Sciences, Beijing, 100730 China

**Keywords:** Hepatocellular carcinoma, Sorafenib resistance, MenSCs, Combination therapy

## Abstract

**Background:**

Sorafenib is a first-line drug targeting the RTK-MAPK signalling pathway used to treat advanced hepatocellular carcinoma (HCC). However, tumour cells readily develop sorafenib resistance, limiting long-term therapy with this drug. In our previous study, we found that human menstrual blood-derived stem cells (MenSCs) altered the expression of some sorafenib resistance-associated genes in HCC cells. Therefore, we wanted to further explore the feasibility of MenSC-based combination therapy in treating sorafenib-resistant HCC (HCC-SR) cells.

**Methods:**

The therapeutic efficiency of sorafenib was determined using CCK-8 (Cell Counting Kit-8), Annexin V/PI and clone formation assays in vitro and a xenograft mouse model in vivo. DNA methylation was determined using RT‒PCR and methylated DNA immunoprecipitation (MeDIP). Autophagy was detected by measuring LC3-II degradation and autophagosome maturation. Transmission electron microscopy identified autophagosomes and mitochondria. Physiological functions of mitochondria were assessed by measuring the ATP content, reactive oxygen species (ROS) generation, and mitochondrial membrane potential (MMP).

**Results:**

The tumour suppressor genes BCL2 interacting protein 3 (*BNIP3*) and BCL2 interacting protein 3 like (*BNIP3L*) were silenced by promoter methylation and that BNIP3 and BNIP3L levels correlated negatively with sorafenib resistance in HCC-SR cells. Strikingly, MenSCs reversed sorafenib resistance. MenSCs upregulated BNIP3 and BNIP3L expression in HCC-SR cells via tet methylcytosine dioxygenase 2 (TET2)-mediated active demethylation. In HCC-SR cells receiving sorafenib and MenSC combination therapy, pressure from sorafenib and elevated BNIP3 and BNIP3L levels disrupted balanced autophagy. Hyperactivation of mitophagy significantly caused severe mitochondrial dysfunction and eventually led to the autophagic death of HCC-SR cells.

**Conclusions:**

Our research suggests that combining sorafenib and MenSCs may be a potentially new strategy to reverse sorafenib resistance in HCC-SR cells.

**Supplementary Information:**

The online version contains supplementary material available at 10.1186/s13287-023-03278-8.

## Background

Hepatocellular carcinoma (HCC) is one of the most common carcinomas and is the second leading cause of cancer-related death worldwide [1]. Sorafenib, a small-molecule multikinase inhibitor, was the first systematic first-line drug approved for patients with advanced HCC by the Food and Drug Administration [2]. However, tumour cells are susceptible to developing sorafenib resistance, which largely limits its long-term therapeutic efficacy. One common method for delaying the development of drug resistance is combining sorafenib with other small-molecule drugs. However, the side effects of nonspecific accumulation of these small-molecular drugs are also a potential nonnegligible risk in clinical practice. Thus, studies exploring new solutions for reducing sorafenib resistance are important.

Human menstrual blood-derived stem cells (MenSCs) are a new type of mesenchymal stem cell (MSC) [3] that exhibit stable proliferation and considerable potential for self-renewal, differentiation, and immunosuppression [4]. MenSCs can be directly isolated from discharged menstrual fluids without ethical concerns and invasive procedures and are to some extent more readily accessible than MSCs from other regular sources, such as bone marrow and adipose tissues [5, 6]. MenSCs have exhibited efficacy in treating animal models of liver fibrosis [7], premature ovarian failure [8], and intrauterine adhesion [9]. In addition, MenSCs impose little immunogenicity and tumorigenicity after transfer into nude mice [6], and MenSC-based cancer therapy has achieved some progress in vitro and in vivo [10–12].

Our previous research revealed that MenSCs epigenetically modify HCC cells and exert an inhibitory effect on HCC cell growth. We also found that MenSCs altered the expression of some chemotherapy resistance-associated genes, which attracted our interest in exploring whether MenSCs might be a choice for combination therapy with small-molecule drugs. Among these chemotherapy resistance-associated genes, we found that BCL2 interacting protein 3 like (BNIP3L) was significantly upregulated by MenSCs in HCC cells. BNIP3L and its highly homologous gene BCL2 interacting protein 3 (BNIP3) are mitochondrial outer membrane proteins that are members of the BH3-only protein subfamily in the BCL2 family; they have been reported to function as tumour suppressor genes, and BNIP3 was reported to be silenced by DNA methylation in HCC cells during the development of sorafenib resistance, which intrigued us. Generally, DNA demethylation is classified into three major pathways: (1) active DNA methylation mediated by the ten-eleven translocation family (tet methylcytosine dioxygenase 1, 2, and 3 (TET1, TET2, and TET3)), (2) de novo DNA methylation, and (3) passive demethylation depending on the DNA methyltransferase family (DNA methyltransferases 1, 3 alpha, and 3 beta (DNMT1, DNMT3A, and DNMT3B))/replication (13). In this study, we further determined that BNIP3 and BNIP3L levels were negatively correlated with sorafenib resistance in sorafenib-resistant HCC (HCC-SR) cells due to promoter hypermethylation and found that MenSCs restored BNIP3 and BNIP3L expression in HCC-SR cells mainly through TET2-mediated active demethylation. Combination therapy with sorafenib and MenSCs induced the hyperactivation of mitophagy, which in turn led to the autophagic cell death of HCC-SR cells. In summary, this work further explores the feasibility of MenSCs in the field of tumour therapy and presents a new strategy for reversing sorafenib resistance.

## Methods

### Cell culture and induction of hypoxic conditions

Huh7 cells (hepatocellular carcinoma) were from the National Collection of Authenticated Cell Cultures (NCACC, Shanghai, China). HepG2 cells (hepatocellular carcinoma) were from the American Type Culture Collection (ATCC, Manassas, VA, USA). Hepatocellular carcinoma liver metastasis three times (HCCLM3) cells (hepatocellular carcinoma) were obtained from the Division of Hepatobiliary and Pancreatic Surgery, Department of Surgery (First Affiliated Hospital, School of Medicine, Zhejiang University, Hangzhou, China). Cells were cultured in DMEM medium (Thermo Fisher Scientific, Waltham, MA, USA) supplemented with 10% fetal bovine serum (Thermo Fisher Scientific) in 5% CO_2_ at 37 °C.

In this study, MenSCs were used at passage ≤ 8 in vitro and 5 in vivo; these cells were provided by Innovative Precision Medicine Group (IPM, Hangzhou, China) and then identified and cultured as previously described [14]. Briefly, surface markers of MenSCs were determined by performing flow cytometry analyses, and MenSCs should be positive for MSC surface markers CD29, CD73, CD90 and CD105 and negative for CD34, CD45, CD117, and human leukocyte antigen DR (HLA-DR). Antibodies against CD29 (#561795), CD73 (#561014), CD90 (#561970), CD105 (#560839), CD34 (#560941), CD45 (#560975), CD117 (#561682), and HLA-DR (#560943) were purchased from BD Biosciences (Franklin Lakes, NJ, USA) and be used according to the manufacturer's instructions. The differentiation potential of MenSCs was identified using trilineage differentiation kits according to the manufacturer's instructions (Cyagen, Santa Clara, CA 95050, USA). The images of trilineage differentiation were captured using an OLYMPUS IX83-DP70 fluorescence microscopy (Olympus Corporation, Tokyo, Japan) and CellSens Standard acquisition software (Olympus Corporation, Tokyo, Japan). The resolution of each image is 1360 × 1024. MenSCs were cultured in α-MEM (Thermo Fisher Scientific) supplemented with 10% fetal bovine serum (Thermo Fisher Scientific) in 5% CO_2_ at 37 °C.

As BNIP3/BNIP3L are mainly transcribed by HIF-1α under hypoxic conditions, some experiments were performed under hypoxic conditions. Hypoxic conditions were created in a hypoxic chamber (Mitsubishi Gas Chemical Company, Inc., Japan) containing 1% O_2_ at 37 °C.

### Measurement of cell viability

Cell viability was detected using a CCK-8 colorimetric kit (Beyotime, Shang Hai, China) according to the manufacturer's instructions.

### Construction of sorafenib-resistant cell lines

HCC cells were cultured in complete DMEM supplemented with escalating doses of sorafenib (MedChemExpress, Monmouth Junction, NJ, USA) for 12 months to establish sorafenib-resistant HCC cells as previously described [15]. Sorafenib was dissolved in DMSO (Sigma‒Aldrich, Merck, Darmstadt, Germany), and the final DMSO concentration was ≤ 0.1%. Specifically, the starting concentration of sorafenib was 0.5 µM and gradually increased to 6 µM (increased 0.125 µM per week, up to 6 µM). Parental cell lines were cultured in complete DMEM supplemented with 0.1% DMSO and served as controls. The half maximal inhibitory concentration (IC50) at 48 h was used as a metric to evaluate the extent of HCC cell resistance to sorafenib, which was calculated using GraphPad Prism 9 software (GraphPad, San Diego, CA) according to the results of the CCK-8 analysis. The IC50 of a successfully constructed sorafenib-resistant HCC line should be ≥ 2 times that of their parental cell line.

### Coculture of MenSCs with HCC cells

MenSCs were plated on the 0.4 μm permeable polyester membrane in Transwell supports (Corning, Wiesbaden, Germany). HCC cells were seeded onto 6-well or 12-well plates (Corning) on the same day. Transwell inserts containing MenSCs were placed above the wells seeded with HCC cells on the next day. For cells in other groups that did not undergo coculture, permeable transwell supports without MenSCs were placed above the wells, and an equal amount of medium was added as a coculture control. For sorafenib-related experiments, cells in other groups that were not treated with sorafenib were cultured in complete DMEM supplemented with 0.1% DMSO as a medium control.

The densities of MenSCs and HCC cells used for the different experiments are listed in Additional file 3, Table 1.

### Animal work

Healthy BALB/c nude mice (4 weeks old, males) obtained from SLAC laboratory Animal Co., Ltd. (Shanghai, China) were maintained at the Laboratory Animal Center of Zhejiang University (ZJU-LAC) and strictly followed the instructions by Laboratory Animal Welfare and Ethics Committee of Zhejiang University (ZJU-IACUC). All of our animal experiments were approved by the Ethics Committee of ZJU-IACUC, and the ethics code is ZJU20210278. All animals were housed in the same specific pathogen-free (SPF) room and were provided with a constant temperature, 50% humidity, a 12-h cycle of light and dark, and plenty of water and food to avoid potential environmental confounders. Each animal was considered one experimental unit.

Sorafenib-resistant xenograft tumours were established to assess the effect of the combination therapy in vivo by referring to a previous study [16]. Briefly, Mice (*n* = 48) were subcutaneously injected with 2 × 10^6^ HCCLM3-SR (*n* = 24) or Huh7-SR cells (*n* = 24) in 50% Matrigel (Corning) to establish xenograft mouse model. The oral vehicle mixtures for sorafenib in animal experiments were water, 95% ethanol, and Cremophor (Sigma‒Aldrich) at a ratio of 6:1:1. From day 7 to day 20, the mice were treated with a low concentration of sorafenib (10–20 mg/kg, p.o., qd) to establish sorafenib-resistant animal models. On day 20, mice-bearing subcutaneous tumours were randomly divided into four treatment groups (*n* = 6, based on previously studies) according to a random number table generated by Excel (Microsoft Corporation, Redmond, WA): negative control (NC), MenSCs, sorafenib, and combination therapy (sorafenib + MenSCs). All animals were included in the subsequent work according to the inclusion criteria that tumour volume ≥ 150 mm^3^. Afterwards, the sorafenib and combination therapy groups were administered sorafenib (50 mg/kg/day), while the NC and MenSC groups received the same volume of the oral vehicle mixtures as vehicle control. The MenSC and combination therapy groups were injected with MenSCs (8 × 10^5^ in 200 µl of PBS, tail vein injection) every 5 days for a total of four injections (the total dose of MenSCs was 3.2 × 10^6^ cells), while the negative control and sorafenib groups were injected with PBS on the same day as control. The tumour volume (mm^3^) was calculated using the formula (*L* × *S*^2^)/2, where *L* represents the long axis of tumours and S represents the short axis of tumours. L and S were recorded every 5 d. Mice were euthanized and excluded once the largest diameter of xenograft tumour was ≥ 2.0 cm, tumours became ulcerated, or showed continuous discomfort (no animal was excluded in advance under these humane endpoints). Mice were euthanized with CO_2_ (at a rate of 30–70% replacement/min in a 10 L chamber for at least 10 min) on day 40, and the tumours were excised for further analysis after all mice had lost consciousness. Researchers analyzing the data were blinded to the group allocation until all the statistical results were finally obtained.

### Statistical analysis

Statistical analyses were performed using GraphPad Prism 9 software (GraphPad, San Diego, CA). For statistical comparisons of two groups, Student’s t test was performed. For the comparison of three or more groups, one-way analysis of variance (ANOVA) was performed, and Tukey’s post hoc test was used for multiple comparisons. The data from in vitro assays are presented as the means ± S.E.M. from at least three independent experiments; for in vivo assays, tumour volume and tumour weights are presented as the means ± S.E.M. calculated from the groups. *P* values < 0.05 were considered statistically significant and *P* values ≥ 0.05 were considered nonsignificant. All test information and values supporting the graphs are available in tables in Additional file 5.

Additional information on the methods is provided in Additional file 4 and has also been described previously [17, 18]. Full-length blots are presented in Additional file 6.

## Results

### BNIP3 and BNIP3L levels are negatively correlated with sorafenib resistance in HCC-SR cells

**Fig. 1:**
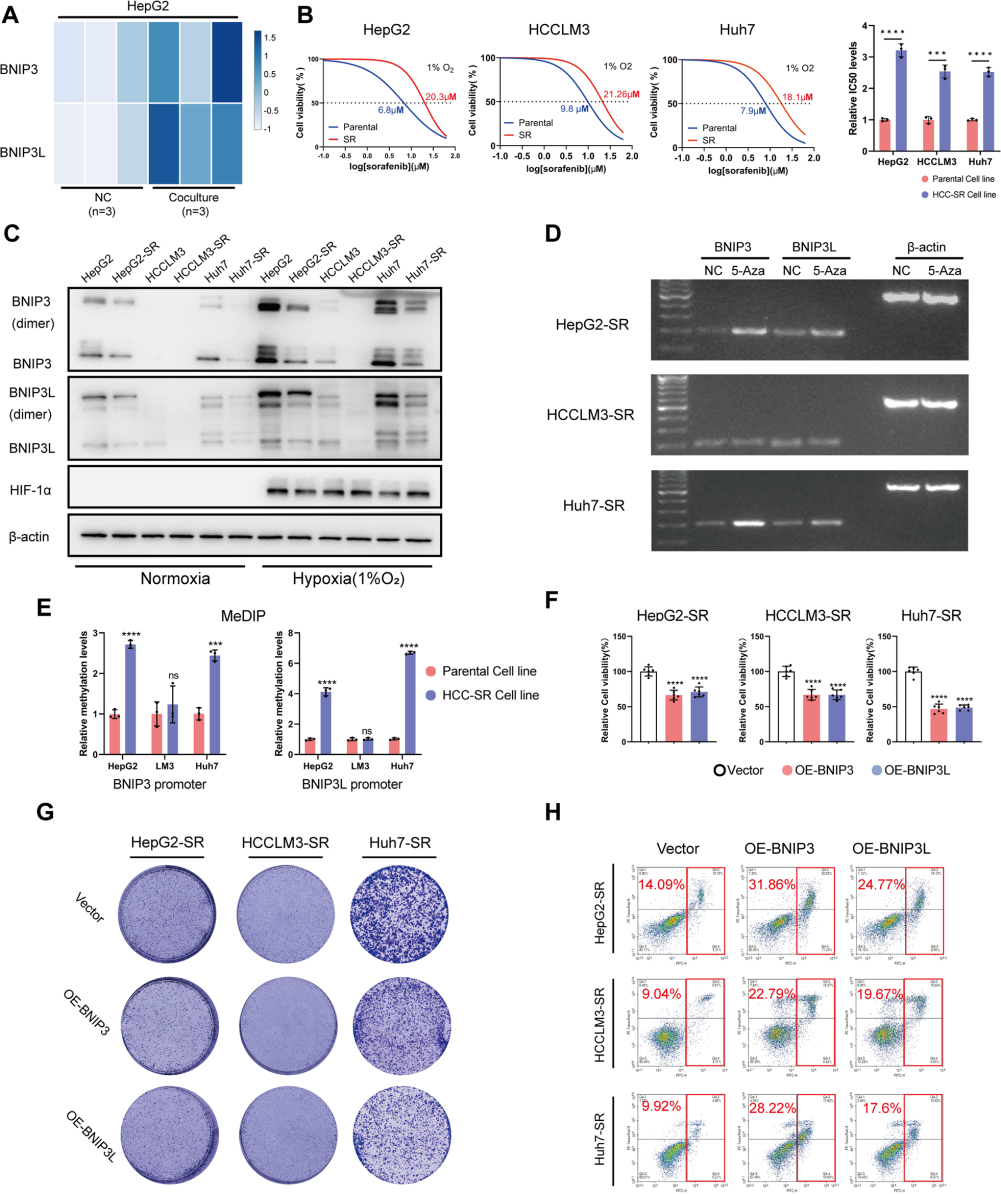
BNIP3 and BNIP3L levels are negatively correlated with sorafenib resistance in HCC-SR cells. **A** Heatmap of *BNIP3* and *BNIP3L* mRNA expression in HepG2 in response to coculture with MenSCs for 72 h. **B** Half maximal inhibitory concentration (IC50) at 48 h was determined using CCK8 analysis (left, Mean, *n* = 6). Statistical analysis of relative IC 50 levels (right). **C** The levels of BNIP3, BNIP3L, and β-actin were determined using immunoblotting analysis 48 h after cells were cultured under normoxia or hypoxia (1%O_2_) as indicated. **D** Transcription levels of *BNIP3* and *BNIP3L* in HCC-SR cells were semi-quantified by RT-PCR analysis (27 PCR amplification cycles in HepG2-SR and Huh7-SR, 30 PCR amplification cycles in HCCLM3-SR). Cells were treated with 5 µM 5-aza-2-deoxycytidine (5-Aza) for 1 week and cultured under hypoxia for 48 h before harvest. Negative control (NC) group: treated without 5-Aza, other cultural conditions were the same as the 5-Aza group. **E** The 5-mc levels of *BNIP3* and *BNIP3L* promoter regions in HCC cells were determined using qRT-PCR followed with methylated DNA immunoprecipitation (MeDIP). **F** Cell viability was determined using CCK-8 analysis 48 h after cells were treated as indicated. Cells were treated with 10 µM sorafenib under hypoxia after the levels of BNIP3 or BNIP3L were modulated as indicated. **G** Proliferation ability of HCC-SR cells was determined using clone formation assay. Cells were cultured in complete DMEM medium containing 6 µM sorafenib for 1 week after the levels of BNIP3 or BNIP3L were modulated as indicated. **H** Cell viability of HCC-SR cells was determined using Annexin V/PI analysis. Cells were treated with 10 µM sorafenib for 48 h under hypoxia after the levels of BNIP3 or BNIP3L were modulated as indicated. ***p < 0.001, ****p < 0.0001. ns represents not statistically significant. Full-length blots/gels are presented in Additional file 6

As shown in our previous study, MenSCs inhibit HCC growth via the genome-wide alteration of DNA hydroxymethylation and methylation [19]. As MenSCs also altered the expression of some chemotherapy resistance-associated genes, we wanted to further explore the feasibility of MenSC-based combination therapy, and we used sorafenib, the first-line drug for the treatment of advanced HCC. We thus investigated the list of significantly changed genes (fold change > 2, *p* < 0.05) detected using RNA-seq (GSE120160) in our previous study to determine whether MenSCs regulate the expression of sorafenib resistance-related genes that have been reported in the literature [19, 20]. We observed significantly increased *BNIP3L* mRNA levels (fold change = 3.45, *p* = 0.007) in HepG2 cells after MenSC therapy. BNIP3L is a BH3 domain-only protein with 56% sequence homology to BNIP3, and BNIP3 has been reported to correlate with sorafenib resistance [21]. Furthermore, *BNIP3* mRNA levels were also elevated after MenSC therapy (fold change = 2.14), but the difference was not statistically significant (*p* = 0.08) (Fig. 1A, Additional file 1A). However, we noted that this statistical result for *BNIP3* expression contained an outlier (M2-RNA) in the MenSC coculture group (Additional file 1A), suggesting that nonsignificant statistical results for *BNIP3* mRNA levels possibly arose from detection errors using RNA-seq. Thus, we propose that the regulation of BNIP3 expression by MenSCs is also worthy of validation in subsequent experiments (Additional file 3).

We validated the relationship between BNIP3/BNIP3L expression and sorafenib resistance in HCC-SR cells by constructing three HCC-SR cell lines (HepG2-SR, HCCLM3-SR, and Huh7-SR). CCK-8 analysis was performed to detect the half maximal inhibitory concentration (IC50) of sorafenib at 48 h to measure the extent of sorafenib resistance in HCC cells. Compared to corresponding parental cells, a significant increase in the IC50 of sorafenib in HCC-SR cells reflected a remarkable decrease in sensitivity to sorafenib in HCC cells, suggesting that we successfully construed sorafenib-resistant HCC cell lines (Fig. 1B). A decrease in BNIP3 levels in HepG2-SR cells has been reported [20], but researchers have not yet clearly determined how BNIP3L levels change; therefore, we measured the BNIP3 and BNIP3L protein levels in our HCC-SR cells using immunoblotting. Half of the samples were collected after incubation under hypoxic conditions (1% O_2_) because BNIP3/BNIP3L are mainly transcribed by HIF-1α [22]. The results verified that BNIP3 and BNIP3L levels were reduced in HCC-SR cells, particularly in HepG2-SR and Huh7-SR cells (Fig. 1C, Additional file 1C). Both the *BNIP3* and *BNIP3L* promoter regions contain CpG islands, and downregulation of BNIP3 and BNIP3L is always accompanied by promoter methylation in different cancer types [23, 24]. Therefore, we further explored whether promoter hypermethylation was responsible for the reduction in BNIP3 and BNIP3L levels in HCC-SR cells by detecting the levels of the *BNIP3* and *BNIP3L* transcripts after demethylation [25]. 5-Aza-deoxycytidine (5-Aza) was used as a DNA methyltransferase inhibitor to induce DNA demethylation [26]. Levels of the *BNIP3* and *BNIP3L* mRNAs in HepG2-SR and Huh7-SR cells were increased under hypoxic conditions after DNA demethylation was induced by 5-Aza compared to the negative control (NC) group. The results indicated that demethylation treatment reactivated the transcription of *BNIP3* and *BNIP3L* under hypoxic conditions, indicating that DNA hypermethylation of the *BNIP3* and *BNIP3L* promoters was responsible for their inhibition in HepG2-SR and Huh7-SR cells (Fig. 1D). These results were further supported by MeDIP-qPCR (methylated DNA immunoprecipitation-qPCR) (Fig. 1E), which suggested that the methylation levels of *BNIP3* and *BNIP3L* promoters were significantly increased in HCC-SR cell lines compared to their corresponding parental cell lines. However, HCCLM3 appeared to be a cell line with low endogenous expression of BNIP3 and BNIP3L. The BNIP3/BNIP3L levels in HCCLM3 cells were much lower than those in HepG2 and Huh7 cells (Fig. 1C, Additional file 1B), and promoter methylation was rarely observed (Fig. 1D–E).

The aforementioned results confirmed that BNIP3 and BNIP3L levels have a negative correlation with sorafenib resistance in some HCC-SR cells.

### Overexpression of BNIP3 and BNIP3L resensitizes HCC-SR cells to sorafenib

Next, we further verified whether the decreased BNIP3 and BNIP3L levels were responsible for the reduced sensitivity of some HCC-SR cells to sorafenib. BNIP3 and BNIP3L were overexpressed in HCC-SR cells, and the therapeutic efficacy of sorafenib was evaluated by performing CCK-8, clone formation and Annexin V/PI assays. A CCK-8 assay was used to detect cell viability, clone formation assays were used to detect cell proliferation, and Annexin V/PI staining was performed to detect apoptotic cells. Compared to the control group (cells transfected with empty vector), HCC-SR cells overexpressing BNIP3 or BNIP3L exhibited reduced viability (Fig. 1F) and proliferation (Fig. 1G) but an increased percentage of apoptotic cells in response to the same sorafenib treatments (Fig. 1H). These results showed that overexpression of BNIP3 and BNIP3L resensitized HCC-SR cells to sorafenib.

### MenSCs reverse sorafenib resistance in HCC-SR cells

**Fig. 2:**
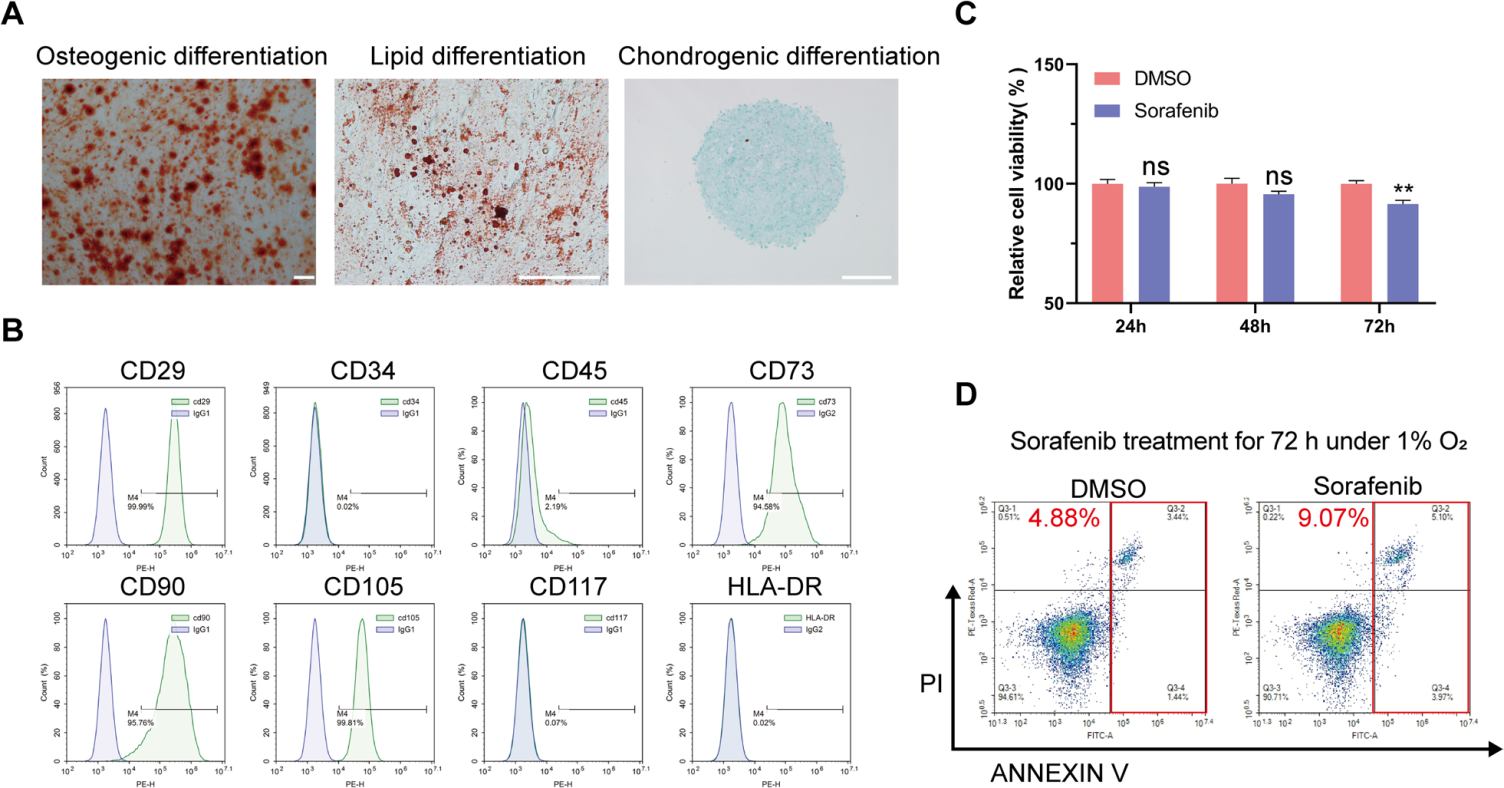
Characterization of MenSCs and cytotoxic effects of sorafenib on MenSCs. **A** Tri-lineage differentiation potential of MenSCs. Scale bar: 100 µm. MenSCs used in this study exhibited good multidifferentiation potential. **B** Surface markers of MenSCs were analyzed by flow cytometry. MenSCs used in this work were positive for MSC surface markers CD29, CD73, CD90 and CD105 and negative for CD34, CD45, CD117, and human leukocyte antigen DR (HLA-DR). **C** Cell viability was determined using CCK-8 analysis after MenSCs were treated with sorafenib for 24, 48, and 72 h. **D** Cell viability was determined using Annexin V/ PI staining 72 h after MenSCs were treated with sorafenib (10 µM) under hypoxia. The results in **C**, **D** suggested that Sorafenib exerted a weak cytotoxic effect on MenSCs. **p < 0.01. ns represents not statistically significant

The immunogenicity and multidifferentiation potential of MenSCs were validated by performing flow cytometry and tri-lineage differentiation assays. These results revealed that the MenSCs used in this study exhibited good multidifferentiation potential and weak immunogenicity and were amenable for transplantation into animals (Fig. 2A, B). We also detected the cytotoxic effects of sorafenib on MenSCs using CCK-8 and Annexin V/PI analyses. Sorafenib exerted a weak cytotoxic effect on MenSCs, which we consider within an acceptable range (Fig. 2C, D).

**Fig. 3:**
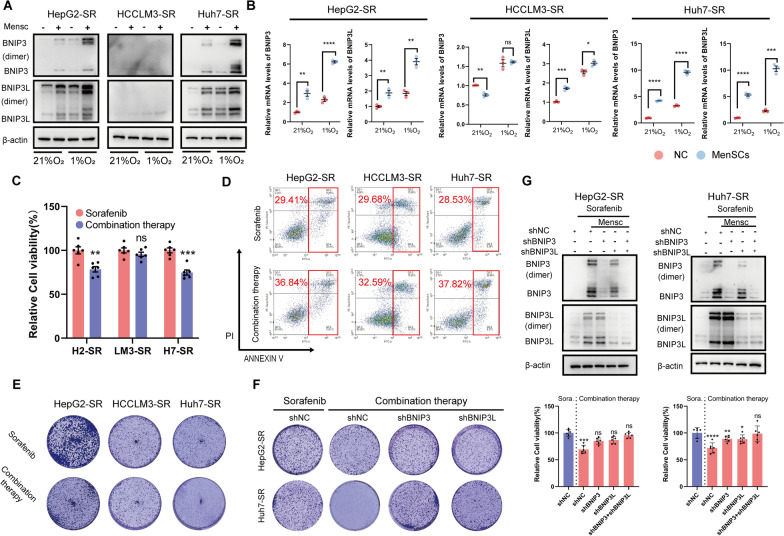
MenSCs reverse sorafenib resistance in HCC-SR cells. **A**, **B** The regulation of BNIP3 and BNIP3L expression by MenSCs in HCC-SR cells was, respectively, determined using immunoblotting (protein) and qRT-PCR (mRNA) analyses. MenSCs significantly upregulated BNIP3 and BNIP3L expression in HepG2-SR and Huh7-SR cells. Cells were cultured under normoxia or hypoxia (1%O_2_) for 48 h. **C**, **D** Cell viability and apoptosis of HCC-SR cells were determined using CCK-8 and Annexin V/PI analyses, respectively. Combination therapy resulted in a further reduction in cell viability (**C**) and an increase in apoptosis (**D**) in HepG2-SR and Huh7-SR cells compared to sorafenib alone. Cells were cultured under hypoxia for 48 h. Sorafenib: 10 µM. **E** Cell proliferation of HCC-SR cells was determined using clone formation assay. Combination therapy resulted in a further reduction in cell proliferation compared to sorafenib alone. **F** Cell proliferation ability was determined using clone formation assay after the levels of BNIP3 or BNIP3L in HCC-SR cells were modulated as indicated. Knocking down BNIP3 or BNIP3L rescued the decreased proliferation observed after combination therapy in HepG2-SR and Huh7-SR cells. Cells in **E**, **F** were cultured in complete DMEM medium containing 6 µM sorafenib for one week; and cells receiving combination therapy were cocultured with MenSCs during the test. **G** Cell viability was determined using CCK-8 analysis after the levels of BNIP3 and BNIP3L in HCC-SR cells were modulated as indicated. Knocking down BNIP3 and BNIP3L rescued the decreased cell viability observed after combination therapy in HepG2-SR and Huh7-SR cells. Cells were cultured under hypoxia for 48 h. Sorafenib: 10 µM. *p < 0.05, **p < 0.01, ***p < 0.001, ****p < 0.0001. ns represents not statistically significant. Full-length blots are presented in Additional file 6

Next, we detected the regulation of BNIP3 and BNIP3L expression by MenSCs in HCC-SR cells using immunoblotting and qRT‒PCR analyses. Cells were collected after coculture with MenSCs under normoxic or hypoxic conditions. MenSCs significantly upregulated BNIP3 and BNIP3L expression in HepG2-SR and Huh7-SR cells. However, the expression of BNIP3/BNIP3L in HCCLM3-SR cells was not restored by MenSCs (Fig. 3A, B). Since we confirmed the negative relationship between BNIP3/BNIP3L levels and sorafenib sensitivity in the last section, we subsequently detected whether combining sorafenib with MenSCs would resensitize HCC-SR cells to sorafenib. The viability, proliferation and percentage of apoptotic cells were measured by performing CCK-8 assays, clone formation assays, and Annexin V/PI staining, respectively. Combination therapy resulted in a further reduction in cell viability (Fig. 3C) and proliferation (Fig. 3E) and an increase in apoptosis (Fig. 3D) in HepG2-SR and Huh7-SR cells compared to sorafenib alone. Based on these results, the combination therapy restored the sensitivity of HepG2-SR and HuH7-SR cells to sorafenib. However, these changes induced by the combination therapy were rarely observed in HCCLM3-SR cells (low endogenous expression of BNIP3/BNIP3L). Then, rescue experiments were performed to determine whether BNIP3 and BNIP3L play a role in the resensitization induced by MenSCs. The results of clone formation assays suggested that knocking down BNIP3 or BNIP3L rescued the decreased proliferation observed after combination therapy (Fig. 3F), and the cell viability analysis produced similar results (Fig. 3G). Therefore, BNIP3 and BNIP3L play important roles in the ability of MenSCs to reverse sorafenib resistance.

Taken together, MenSCs reversed sorafenib resistance in some HCC-SR cells by restoring BNIP3 and BNIP3L expression.

### Combination therapy promotes autophagy flux in Huh7-SR cells

**Fig. 4:**
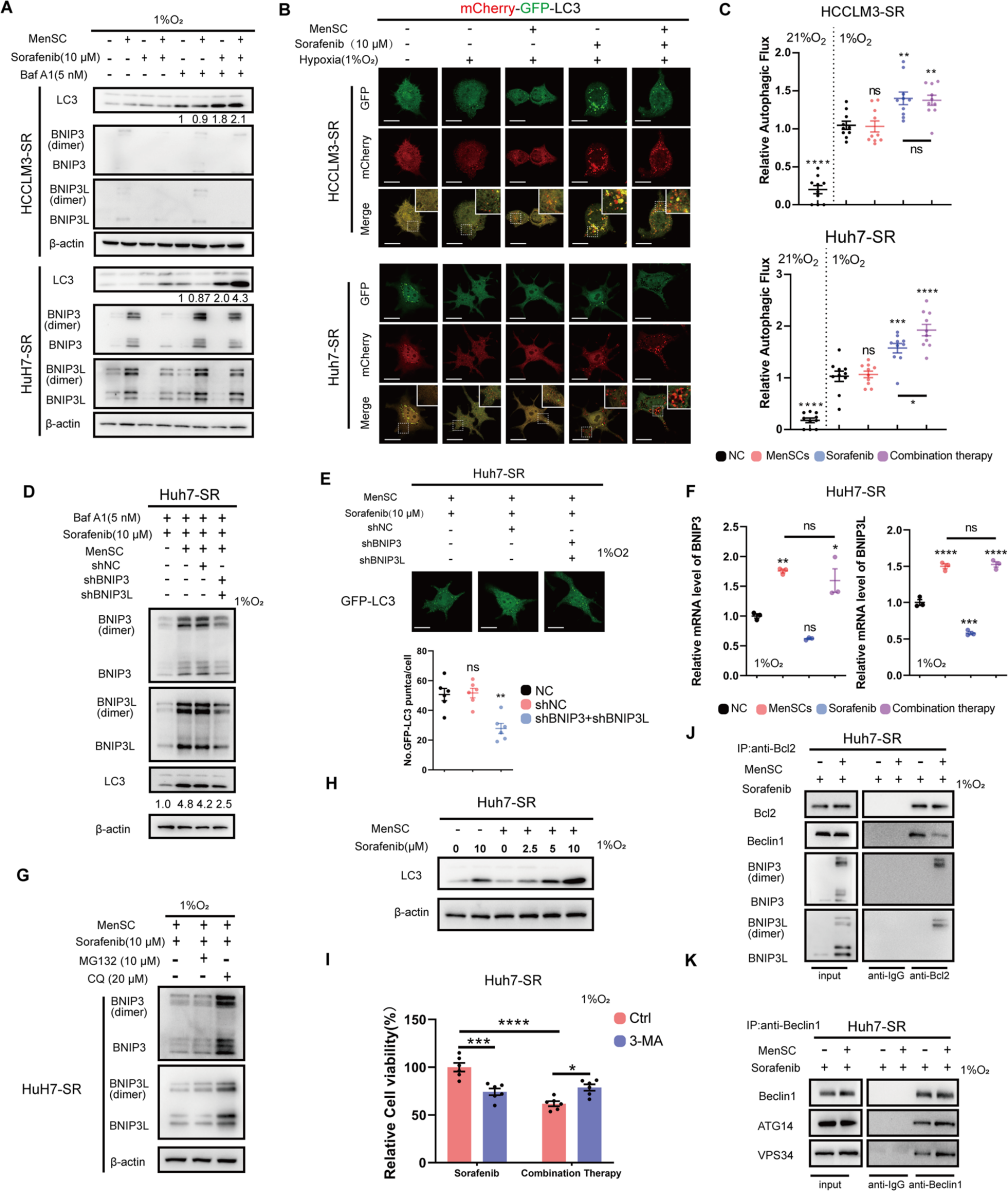
Combination therapy switches protective autophagy to autophagic cell death in Huh7-SR cells. **A** The levels of BNIP3, BNIP3L, LC3, and β-actin were determined using immunoblotting analysis 48 h after cells were treated as indicated. The ratio of LC3-II/LC3-I was labelled below the LC3 lane. BafA1 was added 12 h before harvest. **B**, **C** The expression of mCherry-GFP-LC3 was determined by confocal microscopy analysis 48 h after cells were treated as indicated. mCherry^+^ GFP^−^ and mCherry^+^ GFP^+^ puncta were quantified and summarized to determine the relative autophagy flux. Scale bar: 5 µm. **D** The levels of BNIP3, BNIP3L, LC3, and β-actin in Huh7-SR cells were determined using immunoblotting analysis. Cells were administered sorafenib or combination therapy under hypoxia for 48 h after the levels of BNIP3 and BNIP3L were modulated as indicated. BafA1 was added 12 h before harvest. **E** The expression of GFP-LC3 was determined by confocal microscopy analysis. Cells were administered combination therapy under hypoxia for 48 h. Scale bar: 5 µm. **F** The mRNA levels of *BNIP3* and *BNIP3L* were determined using qRT-PCR 48 h after cells were treated as indicated. **G** The levels of BNIP3, BNIP3L, and β-actin were determined using immunoblotting analysis 48 h after Huh7-SR cells were treated as indicated. Cells were treated with MG132 (10 μM) or CQ (20 μM) 6 h before harvest. **H** The levels of LC3 and β-actin were determined using immunoblotting analysis 48 h after Huh7-SR cells were treated as indicated. **I** Cell viability was determined using CCK-8 analysis 48 h after Huh7-SR cells were treated as indicated. 3-MA was added 24 h before harvest. **J–K** The interactions between BCL2 and Beclin1/BNIP3/BNIP3L (**J**) or Beclin1 and ATG14/VPS34 (**K**) in Huh7-SR cells were determined using immunoblotting analysis followed with co-IP 48 h after indicated treatments. *p < 0.05, **p < 0.01, ***p < 0.001, ****p < 0.0001. ns represents not statistically significant. Full-length blots are presented in Additional file 6.

In the previous sections, we found that sorafenib resistance was reversed by MenSC-based combination therapy by upregulating BNIP3 and BNIP3L. Both sorafenib and BNIP3/BNIP3L were reported to be associated with autophagy, and we hypothesized that they might be linked and analyzed autophagy [27, 28]. Autophagy flux is a well-established indicator of autophagic activity [29]. An LC3-II degradation analysis and GFP-LC3 assays were performed to analyze the change in the autophagy flux of HCC-SR cells overexpressing BNIP3 and BNIP3L, and the results suggested that overexpressing BNIP3 and BNIP3L enhanced the autophagy flux of HCC-SR cells (Additional file 2A, B). Next, we measured the effects of sorafenib and MenSCs on autophagy flux in HCCLM3-SR and Huh7-SR cells using the LC3-II degradation assay and the autophagosome maturation assay (see the additional Methods section (Additional file 4) for details). HCCLM3-SR cells served as a negative control for combination therapy. Sorafenib increased autophagy flux in HCC-SR cells, and combination therapy further enhanced autophagy flux in Huh7-SR cells but did not alter this process in HCCLM3-SR cells (Fig. 4A–C). We used shRNAs targeting BNIP3 and BNIP3L to perform a rescue assay and investigate whether BNIP3 and BNIP3L are responsible for the difference in autophagy flux between HCCLM3-SR and Huh7-SR cells after the administration of the combination therapy. The increase in autophagy flux observed in Huh7-SR cells receiving combination therapy was rescued by knocking down BNIP3 and BNIP3L, indicating that the above-mentioned discrepancy was indeed caused by restored BNIP3 and BNIP3L expression (Fig. 4D, E).

### Restoration of BNIP3 and BNIP3Lis directly involved in the autophagy process

We noted that in Huh7-SR cells, the difference in BNIP3/BNIP3L protein levels between the MenSC group and the combination therapy group (Fig. 4A, Huh7-SR, lane 2 vs. lane 4) was eliminated by bafilomycin A1 (BafA1, a lysosome inhibitor) treatment (Fig. 4A, Huh7-SR, lane 6 vs. lane 8), which indicated that BNIP3 and BNIP3L were directly involved in autophagy. The *BNIP3* and *BNIP3L* mRNA levels in Huh7-SR cells were subsequently measured using qRT‒PCR to confirm the aforementioned finding, and the results indicated that BNIP3 and BNIP3L were partially degraded rather than downregulated in Huh7-SR cells from the combination therapy group compared to the MenSC group (Fig. 4F). Moreover, this degradation was blocked by chloroquine (CQ, another lysosome inhibitor) but not MG132 (a proteasome inhibitor), suggesting that BNIP3 and BNIP3L were directly engaged in autophagy and degraded by the autophagy‒lysosome pathway in Huh7-SR cells receiving combination therapy (Fig. 4G).

Based on these results, combination therapy promoted autophagy flux in Huh7-SR through autophagy mediated by BNIP3 and BNIP3L.

### Combination therapy switches protective autophagy to autophagic cell death in Huh7-SR cells

In the previous subsection, we found that autophagy flux was promoted by the combination therapy in Huh7-SR cells. However, MenSCs alone did not enhance autophagy flux (Fig. 4A–C). We further compared the autophagy flux of the Huh7-SR cells treated with MenSCs in the presence of a series of sorafenib concentration gradients and reached the same conclusion, revealing that the main stress of autophagy is sorafenib-induced living pressure and that MenSCs did not directly promote autophagy but instead enhanced sorafenib-induced autophagy (Fig. 4H).

We measured cell viability by performing a CCK-8 analysis to explore the effect of autophagy on cell survival. Huh7-SR cells were treated either with sorafenib or combination therapy, and 3-methyladenine (3-MA, an autophagy inhibitor targeting PIK3C3) was used to inhibit the autophagy process. Inhibition of autophagy decreased cell viability in the sorafenib group but increased it in the combination group, indicating that autophagy played a protective role in the sorafenib group while leading to reduced cell viability in the combination group (Fig. 4I).

The relative levels of the interaction between BCL2 and Beclin1 determine autophagy initiation to some extent. BNIP3 and BNIP3L affect autophagic activity mainly by competing with Beclin1 for binding to BCL2. By performing coimmunoprecipitation (co-IP) assays and immunoblotting, we verified that the interactions between Beclin1 and BCL2 in Huh7-SR cells were disrupted after 48 h of coculture with MenSCs, which increased the ability of cells to form autophagy initiation complexes (Beclin1/ Autophagy Related 14 (ATG14)/ Phosphatidylinositol 3-Kinase Catalytic Subunit Type 3 (VPS34)) (Fig. 4J–K).

Taken together, the combination therapy switched sorafenib-induced protective autophagy to autophagic cell death in Huh7-SR cells by hyperactivating autophagy.

### Combination therapy enhances mitochondrial disruption in Huh7-SR cells

**Fig. 5:**
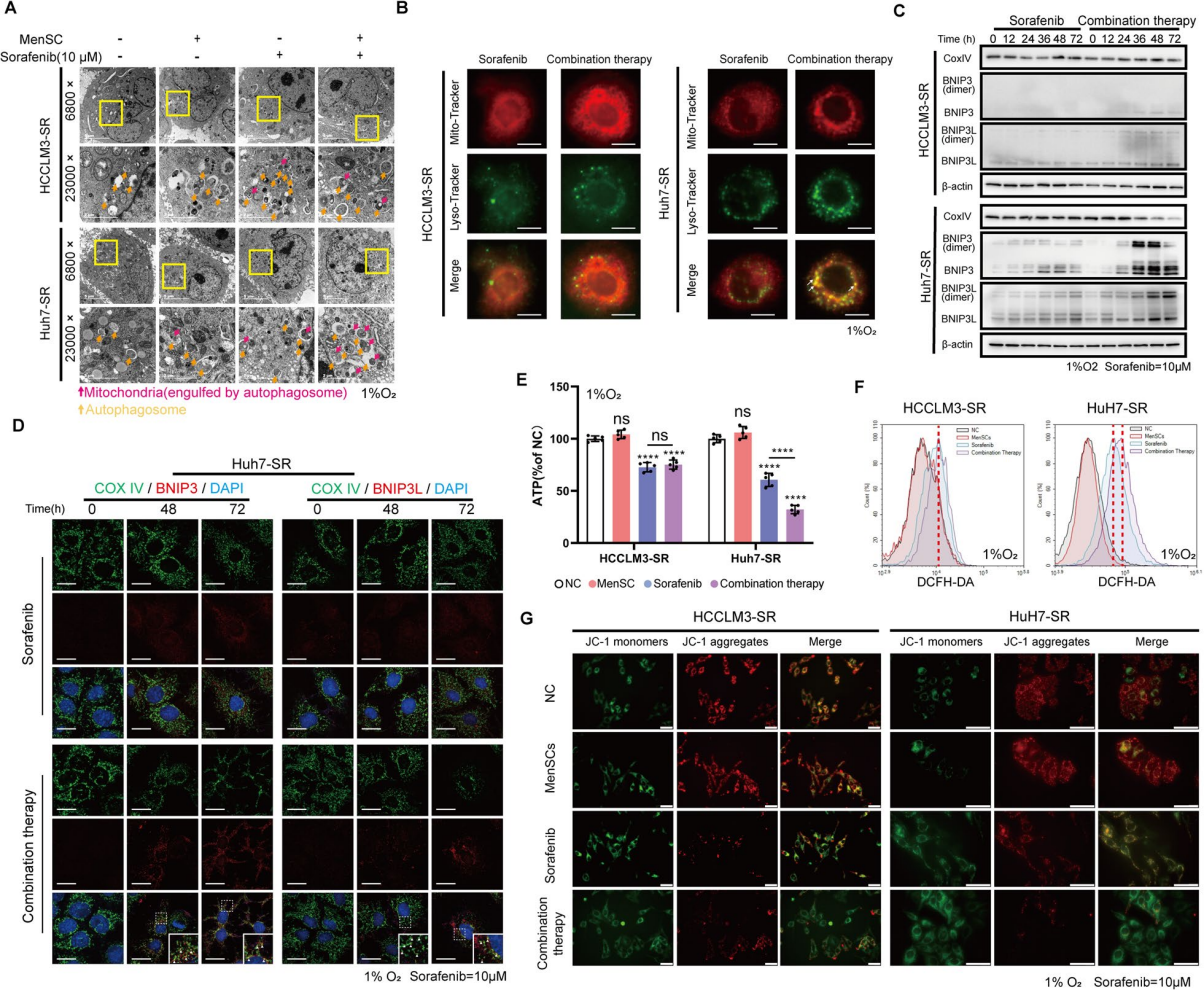
Combination therapy enhances mitochondrial disruption in Huh7-SR cells. **A** Images of autophagosomes and mitochondria in HCC-SR cells were captured by transmission electron microscopy (TEM). Autophagosomes in the Huh7-SR cells receiving combination therapy contained more damaged mitochondria, suggesting an increase in mitophagy. Scale bar: 5 µm for 6800 ×, 2 µm for 23,000 ×. **B** Localizations of mitochondria and lysosomes in HCC-SR cells were observed by microscopy. Mitochondria (red) and lysosomes (green) were, respectively, stained with MitoTracker and LysoTracker. Colocalizations of lysosomes and mitochondria were significantly observed in Huh7-SR cells treated with combination therapy, suggesting mitophagy were activated. Scale bar: 5 µm. **C** The levels of CoxIV, BNIP3, BNIP3L, and β-actin in HCC-SR cells were determined using immunoblotting analysis after cells were treated with sorafenib or combination therapy for 0, 12, 24, 36, 48, and 72 h. The results showed that decreased CoxIV levels (mitochondrial mass) is accompanied by increased BNIP3 and BNIP3L levels over time in the Huh7-SR cells treated with combination therapy. **D** Immunofluorescence staining of BNIP3/BNIP3L (Red) and CoxIV (Green) 0, 48 and 72 h after Huh7-SR cells received sorafenib or combination therapy. Arrows: co-localization of BNIP3 and Cox IV. Scale bar: 10 µm. **E** The ATP content was analyzed, which was further decreased in Huh7-SR cells received combination therapy compared to sorafenib alone. **F** The reactive oxygen species (ROS) levels in HCC-SR cells were measured. Combination therapy caused more accumulation of ROS than sorafenib alone in Huh7-SR cells. **G** Mitochondrial membrane potential (MMP) levels in HCC-SR cells were determined using JC-1 staining. JC-1 aggregates (red) represent the high membrane potential while the JC-1 monomers (green) represent the low membrane potential. The result suggested that combination therapy caused a further decrease in MMP levels in Huh7-SR cells compared to sorafenib alone. Scale bar: 50 µm. Cells in **A**, **B**, and **E**–**G** were treated as indicated for 48 h. ****p < 0.0001. ns represents not statistically significant. Full-length blots are presented in Additional file 6

Next, we performed transmission electron microscopy (TEM) to observe autophagosomes in Huh7-SR cells. TEM images revealed that the autophagosomes in the Huh7-SR cells receiving combination therapy contained more damaged mitochondria, suggesting an increase in mitophagy (Fig. 5A). Enhanced mitophagy was then verified by performing costaining for MitoTracker and LysoTracker, as well as immunoblotting analysis (Fig. 5B, C). We further examined whether BNIP3 and BNIP3L were involved in this mitophagy process by performing immunofluorescence staining to determine the colocalization of BNIP3 and CoxIV (cytochrome c oxidase-IV, mitochondrial marker). As shown in Fig. 5D, confocal images not only confirmed the colocalization of BNIP3 and CoxIV but also showed alterations in mitochondrial morphology in Huh7-SR cells receiving combination therapy. We also measured the ATP content, reactive oxygen species (ROS) generation, and mitochondrial membrane potential (MMP) to assess the physiological function and state of mitochondria. The combination therapy caused more pronounced mitochondrial damage in Huh7-SR cells (Fig. 5E–G). Taken together, these results indicated that the combination therapy led to more severe mitochondrial disruption in Huh7-SR cells through the hyperactivation of mitophagy.

### MenSCs promote BNIP3/BNIP3L expression via TET2-mediated active demethylation in HCC-SR cells

In previous subsections, we verified that MenSCs reversed sorafenib resistance by restoring BNIP3 and BNIP3L expression. However, the mechanism by which MenSCs regulate BNIP3 and BNIP3L expression is unclear. As we have shown that BNIP3 and BNIP3L levels were decreased in HCC-SR cells (Fig. 1C) and that promoter hypermethylation was responsible for the inhibition of *BNIP3* and *BNIP3L* transcription in HCC-SR cells in previous subsections (Fig. 1D, E), we further explored whether these molecules were upregulated by MenSCs in HepG2-SR and Huh7-SR cells through DNA demethylation.

**Fig. 6:**
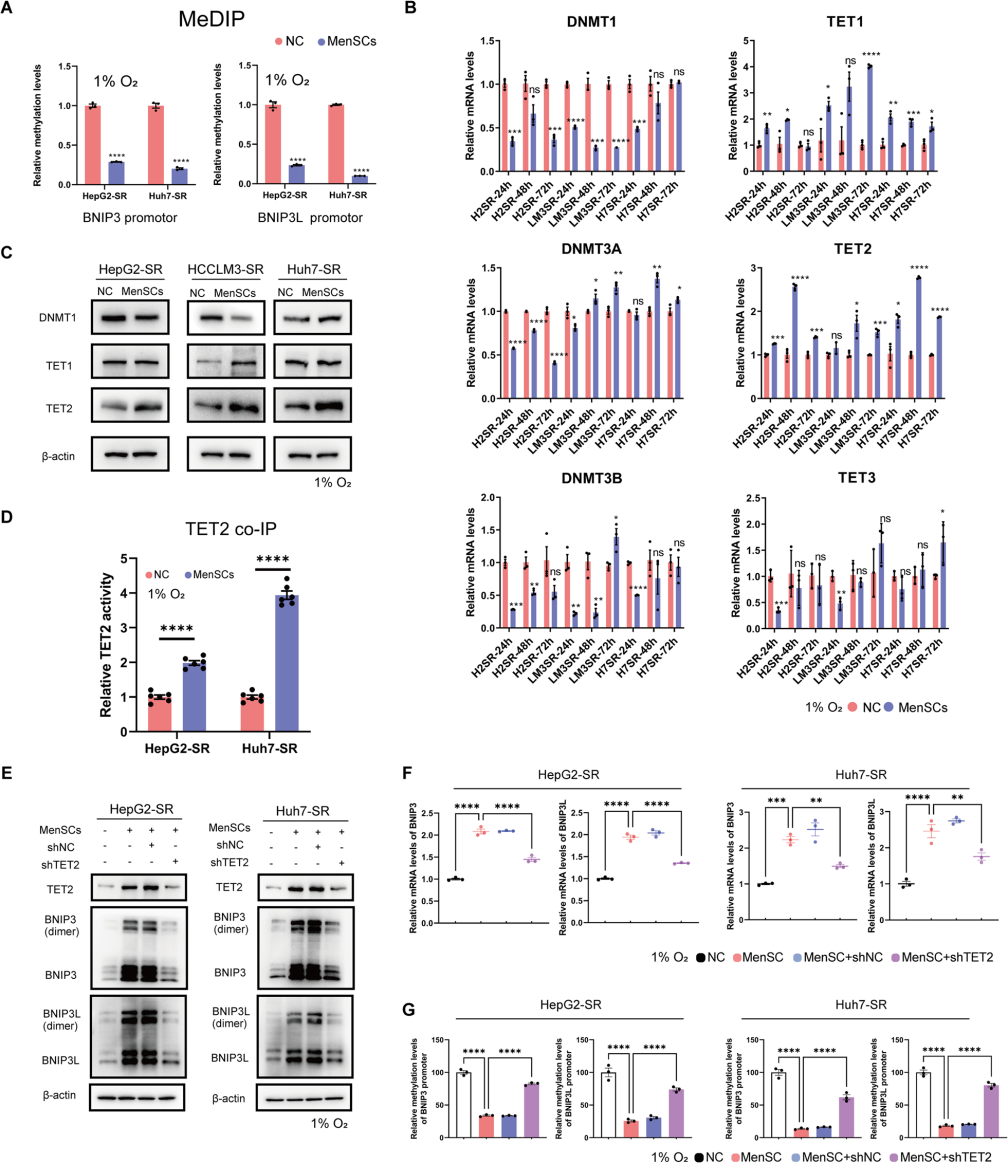
MenSCs restored BNIP3/BNIP3L expression via TET2-mediate active demethylation. **A** The 5-mc levels of *BNIP3* and *BNIP3L* promoter regions in HCC cells were determined using qRT-PCR followed with methylated DNA immunoprecipitation (MeDIP). MenSCs significantly reduced the methylation levels of the *BNIP3* and *BNIP3L* promoters. Cells were cocultured with MenSCs under hypoxia for 48 h. **B** The mRNA levels of *DNMT1*, *DNMT3A*, *DNMT3B*, *TET1*, *TET2*, and *TET3* in HCC-SR cells were determined using qRT-PCR analysis after 24, 48, and 72 h of coculture with MenSCs under hypoxia. **C** The levels of DNMT1, TET1, and TET2 in HCC-SR cells were determined using immunoblotting analysis after 48 h of coculture with MenSCs under hypoxia. According to **B** and **C**, the changes in TET2 transcription levels and protein levels appeared to be the most conserved between HCC-SR cell lines after MenSC treatments, which was stably upregulated by MenSCs in all HCC-SR cell lines. **D** TET2 activity in HCC-SR cells was determined using a TET Hydroxylase Activity Quantification Kit. MenSCs increased TET2 activity in HepG2-SR and Huh7-SR cells. Cells were cocultured with MenSCs under hypoxia for 72 h and 1ug of TET2 protein was purified using co-IP. **E** The levels of TET2, BNIP3, BNIP3L, and β-actin in Huh7-SR cells were determined using immunoblotting analysis.** F** The mRNA levels of *BNIP3* and *BNIP3L* were determined using qRT-PCR analysis.** G** The 5-mc levels of *BNIP3* and *BNIP3L* promoter regions in HCC cells were determined using qRT-PCR followed with methylated DNA immunoprecipitation (MeDIP). The results in **E**–**G** showed that knocking down TET2 rescued both upregulation of BNIP3/BNIP3L levels and decreased methylation levels of *BNIP3* and *BNIP3L* promoters observed in HepG2-SR and Huh7-SR cells received combination therapy. Cells in **E**–**G** were cocultured with MenSCs under hypoxia for 48 h after the levels of TET2 was modulated as indicated. *p < 0.05, **p < 0.01, ***p < 0.001, ****p < 0.0001. ns represents not statistically significant. Full-length blots are presented in Additional file 6

The methylation status of the *BNIP3* and *BNIP3L* promoters in HepG2-SR and Huh7-SR cells was detected using an MeDIP analysis after MenSC treatment, and the results revealed that MenSCs significantly reduced the methylation levels of the *BNIP3* and *BNIP3L* promoters (Fig. 6A). Passive demethylation caused by a decrease in methylases (DNMTs) activity and active demethylation caused by an increase in demethylases (TETs) activity are two of the most common mechanisms regulating demethylation in cells [30]. We quantified the mRNA levels of DNMTs and TETs in HCC-SR cells cocultured with MenSCs to determine how the *BNIP3* and *BNIP3L* promoters were demethylated by MenSCs. Cells were collected after 24, 48, and 72 h of coculture with MenSCs, and a qRT‒PCR analysis was performed. We found that the changes in the mRNA levels of *DNMT1* (decreased), *TET1* (increased), and *TET2* (increased) were more consistent and conserved in all HCC-SR cell lines (Fig. 6B). Based on this result, DNMT1, TET1, and TET2 protein levels were subsequently determined using immunoblotting. Compared to the negative control group (NC), DNMT1 protein levels were decreased in HepG2-SR and HCCLM3-SR cells but slightly increased in Huh7-SR cells upon MenSC treatment; TET1 protein levels were upregulated in HCCLM3-SR cells but not altered in HepG2-SR and Huh7-SR cells after MenSC treatment; and the TET2 protein levels were upregulated by MenSCs in all three HCC-SR cell lines (Fig. 6C). At the mRNA level and protein level, the changes in TET2 appeared to be the most conserved between HCC-SR cell lines after MenSC treatments, which was stably upregulated by MenSCs in all HCC-SR cell lines. We purified the TET2 protein by performing co-IP with a TET2 antibody after cells were cocultured with MenSCs for 72 h to examine whether TET2 was activated in HCC-SR cells after treatment with MenSCs. TET2 activity was then quantified using a TET Hydroxylase Activity Quantification Kit, and we found that MenSCs indeed increased TET2 activity in HepG2-SR and Huh7-SR cells (Fig. 6D).

We verified a role for TET2 in the MenSC-mediated upregulation of BNIP3 and BNIP3L in HepG2-SR and Huh7-SR cells by performing a series of rescue experiments. First, we found that knocking down TET2 significantly rescued the upregulation of BNIP3/BNIP3L levels in HepG2-SR and Huh7-SR cells cocultured with MenSCs, indicating that BNIP3 and BNIP3L were upregulated by MenSCs through TET2 (Fig. 6E). Second, the results of the qRT‒PCR analysis further indicated that knocking down TET2 rescued the increased *BNIP3* and *BNIP3L* mRNA levels in HepG2-SR and Huh7-SR cells cocultured with MenSCs, and thus the regulation of BNIP3 and BNIP3L expression by TET2 was observed at the transcriptional level (Fig. 6F). Finally, the MeDIP analysis directly determined that knockdown of TET2 rescued the decreased methylation levels of the *BNIP3* promoter and *BNIP3L* promoter in HepG2-SR and Huh7-SR cells cocultured with MenSCs, which directly supported the role of TET2 in regulating the decreased methylation levels in the promoter regions of *BNIP3* and *BNIP3L* under coculture conditions (Fig. 6G).

Based on these results, MenSCs increased TET2 activity in HCC-SR cells, and TET2-mediated active demethylation played a dominant role in MenSC-mediated *BNIP3/BNIP3L* promoter demethylation.

### MenSCs enhance the sensitivity of sorafenib-resistant xenograft tumours to sorafenib in vivo

**Fig. 7:**
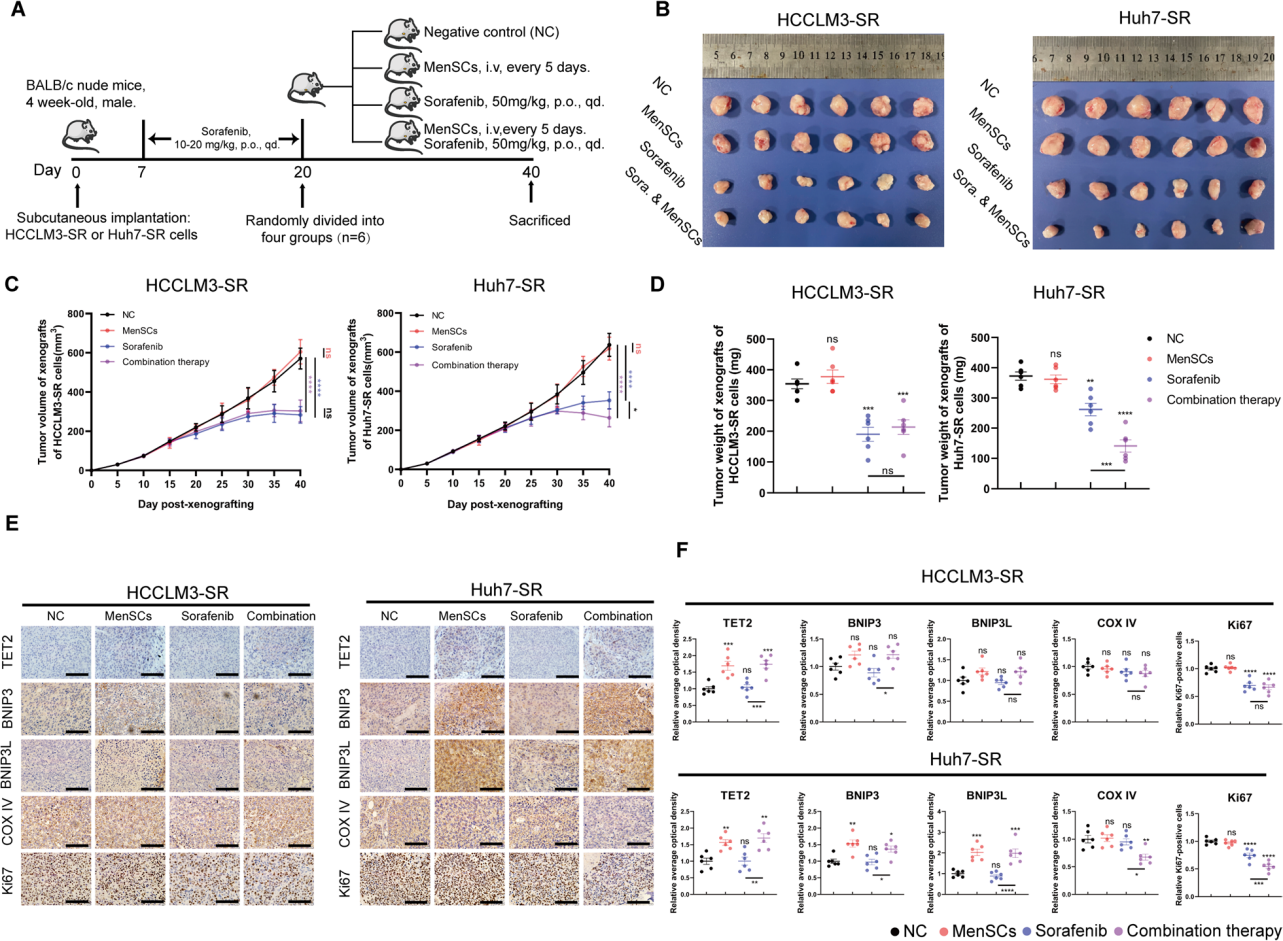
MenSCs enhance the sensitivity of sorafenib to sorafenib-resistant xenograft tumours in vivo. **A** Scheme of the animal experiment (see the Methods section for details). **B** Images of tumours derived from nude mice-bearing xenografts of HCCLM3-SR and Huh7-SR cells (*n* = 6). **C** Line charts depicted tumour growth curves. Tumour volume was measured every 5 d after mice were subcutaneously injected with HCC-SR cells. **D** The weights of tumours were measured after they were excised. According to **C** and **D**, combination therapy caused a further decrease in volume and weight of Huh7-SR xenograft tumours compared to sorafenib alone, which reflected that combination therapy was more effective than sorafenib alone against Huh7-SR xenograft tumours. **E** Immunohistochemical staining of TET2, BNIP3, BNIP3L, Cox IV, and Ki67 in tumours. Scale bar: 100 µm. **F** Relative average optical density of TET2, BNIP3, BNIP3L, COX IV, and relative Ki67-positive cells were measured. MenSC treatment increased the levels of TET2, BNIP3, and BNIP3L in the Huh7-SR xenograft tumours. The mitochondrial mass was determined by CoxIV staining and used to evaluate mitophagy activity. Combination therapy reduced the CoxIV levels in the Huh7-SR xenograft tumours, suggesting that mitophagy was enhanced after the administration of the combination therapy. Ki67 staining was performed to detect the inhibition of cell proliferation. The relative Ki67-positive cells were decreased in Huh7-SR xenograft tumours after the administration of combination therapy, reflecting that the combination therapy exerted a better inhibitory effect on Huh7-SR tumour growth than sorafenib alone (Fig. 6E, F). *p < 0.05, **p < 0.01, ***p < 0.001, ****p < 0.0001. ns represents not statistically significant

Sorafenib-resistant xenograft tumours were established to assess the effect of the combination therapy in vivo (details are described in the Methods section and schematic in Fig. 7A). Consistent with our previous in vitro results, combination therapy was more effective than sorafenib alone against Huh7-SR xenograft tumours, as indicated by a comparison of the tumour weight and volume, but it was not as efficacious against HCCLM3-SR xenograft tumours. In addition, the administration of MenSCs alone neither promoted nor inhibited sorafenib-resistant xenograft tumour growth (Fig. 7B–D). Furthermore, we examined TET2, BNIP3, and BNIP3L protein levels in HCC-SR tumour tissues by performing immunohistochemical staining. MenSC treatment increased the levels of TET2, BNIP3, and BNIP3L in the Huh7-SR xenograft tumours. We determined the number of mitochondria using Cox IV staining to evaluate mitophagy activity. We found that the combination therapy reduced the mitochondrial mass, consistent with our finding that mitophagy was enhanced after the administration of the combination therapy. Moreover, Ki67 staining was performed to detect the inhibition of cell proliferation. The combination therapy exerted a better inhibitory effect on Huh7-SR tumour growth than sorafenib alone (Fig. 7E, F). In conclusion, MenSCs synergized with sorafenib to exert better efficacy in vivo.

## Discussion

Sorafenib remains one of the most effective single-drug treatments for advanced HCC [31]. Based on accumulating evidence, epigenetics, transport processes, cell death regulation, and the tumour microenvironment contribute to sorafenib resistance in HCC [20]. Increased drug doses or combinations with other small-molecule drugs might prolong the survival time to some extent, which is usually accompanied by increased the nonspecific accumulation of drugs. Therefore, new strategies for solving small-molecule drug resistance must be developed.

In previous studies, exogenous MSCs from different origins have been confirmed to home toward tumour sites and inhibit tumour growth animal models [32, 33], and our previous study provided additional epigenetic evidence for elucidating the complicated mechanism of crosstalk between MenSCs and HCC-derived xenograft tumours in the tumour microenvironment [19]. Based on these facts, we further explored whether combining small-molecule drugs and MenSCs might be developed as a new strategy to solve drug resistance. Finally, we determined the feasibility of using the epigenetic modification ability of MenSCs to reverse sorafenib resistance in some HCC cells.

An increasing number of studies have shown that exosomes derived from MSCs, including exosomes derived from MSCs that facilitate chemotherapeutic agents in HCC [34], have potential in treating various diseases in vitro and in vivo. Together with these reports, our work provides a further reference for MSC-based tumour therapy. Of course, there are some differences between our work and theirs. The biggest difference and the innovation of our work is that the most critical part of our work is to explore whether epigenetic regulation caused by MenSCs can resensitize tumours to small-molecule drugs in the tumour microenvironment. This research is continued based on our previous study, and we think that such continuous research can make the previous research more valuable and provide a reference for possible future clinical translation. In our study, MenSCs reversed sorafenib resistance mainly by restoring BNIP3 and BNIP3L expression in HCC-SR cells. BNIP3 and BNIP3L are two BH3 domain-only proteins that have been reported to be associated with cell apoptosis, mitochondrial dysfunction, and mitophagy, and many previous studies have indicated that they are tumour suppressor genes [35–37]. BNIP3 silencing by DNA methylation is a common event in many types of cancers [38, 39], and a clinical investigation suggested that the methylation frequency in the *BNIP3* and *BNIP3L* promoters is associated with a poor prognosis for patients with HCC [40]. BNIP3 silencing tends to occur when cancer cells acquire chemoresistance to drugs, and epigenetic silencing of BNIP3 was also previously observed in sorafenib-resistant cells [21, 41]. Here, our work not only confirmed this phenomenon but also reveals that BNIP3L exhibits the same tendency.

Interestingly, although MenSCs upregulated BNIP3/BNIP3L in Huh7-SR and HepG2-SR cells, MenSCs alone did not directly increase autophagy flux. This finding suggests that BNIP3/BNIP3L compete with Beclin1 for binding to BCL2, allowing cells to form more autophagy initiation complexes but do not directly promote autophagy. In addition, sorafenib itself plays a role in mitochondrial dysfunction, and sorafenib-resistant cells activate a mechanism to reduce mitochondrial biogenesis and circumvent the excessive production of ROS caused by damaged mitochondria, counteracting oxidative stress-induced cell death [42]. In other words, mitochondrial biogenesis and clearance are maintained in a relatively balanced state in HCC-SR cells. When HCC-SR cells are exposed to stress from sorafenib, they use the above-mentioned mechanism to maintain cell survival. However, in cells exposed to combination therapy, this balance was disrupted by the increased BNIP3 and BNIP3L levels, resulting in the hyperactivation of mitophagy and eventually leading to autophagic cell death.

Although we confirmed that MenSCs facilitate a better therapeutic effect of sorafenib, our work still has some limitations. First, most of the data in our research were derived from HCC cell lines but not primary hepatic tumour cells. The main reason is that the genetic background of primary hepatic tumour cells is uncertain and complicated. Thus, we are not sure whether the expression of BNIP3 and BNIP3L in primary hepatic tumour cells from different hosts was the dominant factor causing sorafenib resistance, which may prevent us from drawing stable conclusions. Based on this information, HCC cell lines whose genetic background was relatively well established and had already been used in sorafenib resistance-related studies were selected to conduct our research [15, 42]. During the course of the study, we found that the combination therapy resensitized HepG2-SR and Huh7-SR cells to sorafenib, which was more striking for Huh7-SR cells. However, sorafenib resistance in HCCLM3-SR cells (low endogenous expression of BNIP3/BNIP3L) was not reversed by MenSCs, suggesting that the crosstalk between MSCs and tumour cells is also affected by intertumour heterogeneity. Thus, we used Huh7-SR cells as our main subjects and HCCLM3-SR cells as a negative control. Second, some of our in vitro assays were performed under hypoxic conditions. However, MenSCs and tumour cells may face ischaemic conditions after transplantation in vivo. Sorafenib resistance is influenced by various factors in the tumour microenvironment that are difficult to mimic completely. To our knowledge, BNIP3/BNIP3L are mainly transcribed by HIF-1α [22], and in our study, we established hypoxic conditions that only intended to mimic the transcriptional regulation of BNIP3/BNIP3L by HIF-1α to observe the changes in the expression of BNIP3 and BNIP3L. Moreover, the HIF-1α signalling pathway has also been frequently mentioned in studies assessing the functions of BNIP3 in ischaemia/reperfusion [43], which also suggests to some extent that the expression of BNIP3 and BNIP3L is also mainly regulated by hypoxia under ischaemic conditions. Nevertheless, we acknowledge that some of our experimental designs do not fully mimic the real tumour microenvironment, which must be overcome in the future. Third, in this study, MenSCs are consistent with previous reports, they shown little tumorigenicity both in vivo and in vitro [6]. However, the safety of MSC-based therapies in oncology is still undetermined and debated [44]. Therefore, additional research is needed to enrich our knowledge in this field.

This work also suggests several future directions. First, the mechanism by which MenSCs alter the levels of proteins in the DNMT family and the TET family remains largely unknown. Second, the efficacy of combination therapy is limited by intertumour heterogeneity and encouraged us to conduct more advanced research, such as combining MenSCs with gene-editing technologies to design more targeted therapeutic strategies for tumours with different genetic backgrounds.

## Conclusions

**Fig. 8:**
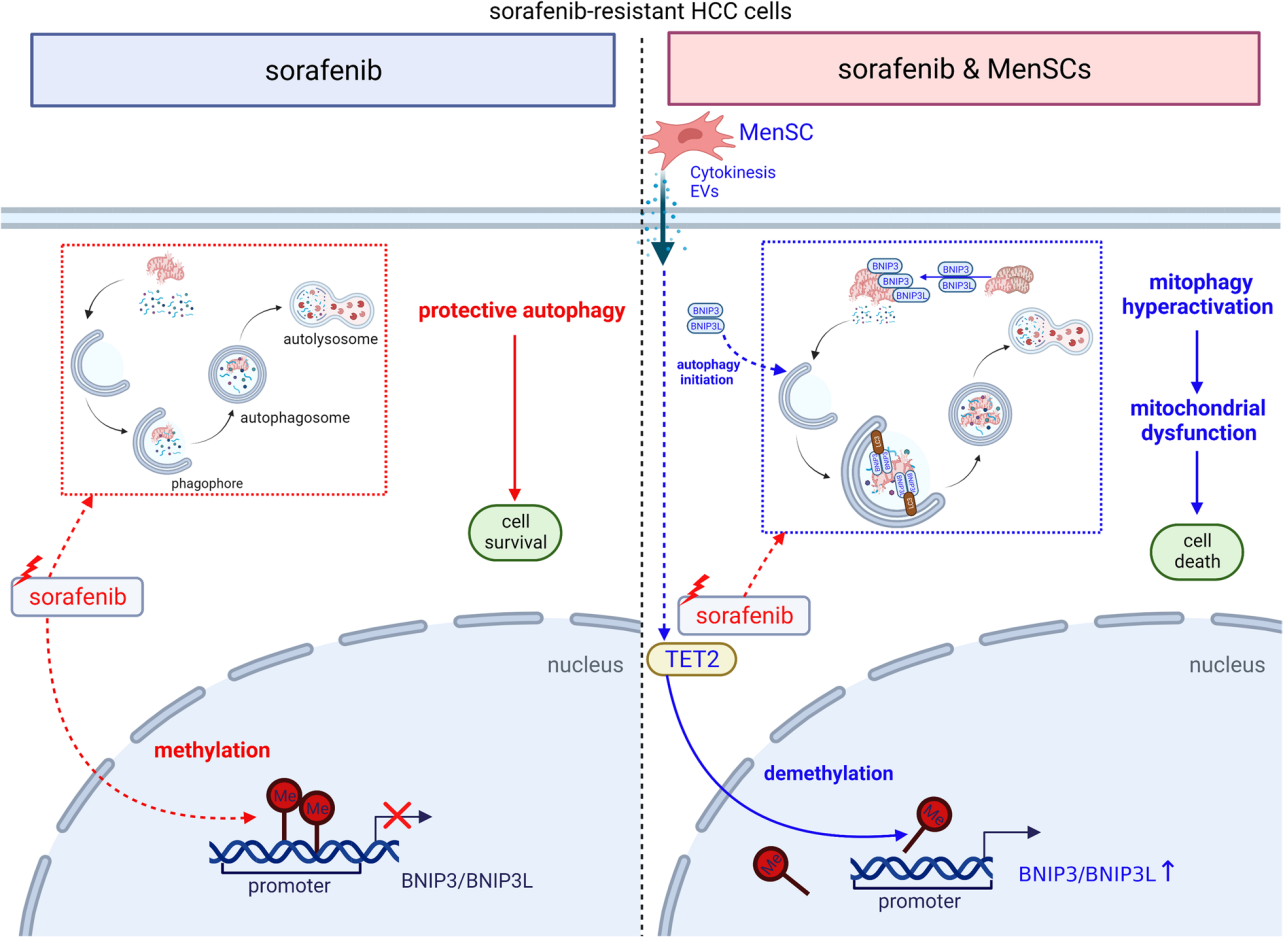
Red: sorafenib-related events. Blue: MenSC-related events. For adaptation to long-term sorafenib treatment, autophagy was activated in HCC-SR cells and maintained a balanced state by removing damaged mitochondria and other autophagic substrates. This kind of autophagy plays a protective role in the tumour progression of HCC-SR cells and helps to maintain sorafenib resistance. In addition, during HCC cell resistance to sorafenib, BNIP3 and BNIP3L were silenced by DNA methylation (left). MenSCs upregulated BNIP3 and BNIP3L expression via TET2-mediated active demethylation. BNIP3 and BNIP3L are involved in promoting autophagic initiation and mitochondrial membrane potential reduction-related mitochondrial dysfunction and directly interact with LC3 to target mitochondria for removal by autophagosomes. When BNIP3 and BNIP3L levels were abundantly restored, the above-mentioned functions of BNIP3 and BNIP3L were also enlarged and induced hyperactivation of mitophagy. Hyperactivation of mitophagy disrupted the balance of protective autophagy in HCC-SR cells, which eventually led HCC-SR cells to undergo autophagic cell death (right). The figure was created with BioRender.com

In summary, our work revealed that combination therapy with sorafenib and MenSCs resensitized HCC-SR cells to sorafenib through mitochondrial dysfunction induced by the hyperactivation of mitophagy (Fig. 8). MenSCs restored BNIP3 and BNIP3L expression in some HCC-SR cells via TET2-mediated active demethylation. Upregulated BNIP3 and BNIP3L disrupt the balance between mitochondrial biogenesis and clearance by competing with Beclin1 for binding to BCL2 in HCC-SR cells, which causes the hyperactivation of mitophagy. Eventually, this hyperactivation of mitophagy leads to mitochondrial dysfunction and induces autophagic cell death. Taken together, our work verified the potential of MenSC-based combination therapy in resensitizing HCC-SR cells to sorafenib, which might provide a new strategy for overcoming sorafenib resistance.

## Supplementary Information

**Additional file 1. Figure S1**. RNA-seq results and characterization of MenSCs. **A** RNA-seq analysis of BNIP3 and BNIP3L mRNA expression in HepG2 in response to coculture with MenSCs for 72 h. C means control group, M means MenSCs coculture group (n=3). **B** The mRNA levels of BNIP3 and BNIP3L were determined using qRT-PCR analysis 48 h after HCC-SR cells were cultured under normoxia or hypoxia (1% O2). **C** The overexposed images of the Immunoblotting analysis of BNIP3 and BNIP3L in Figure 1C. ***p < 0.001, ****p < 0.0001.

**Additional file 2. Figure S2**. Effects of BNIP3 and BNIP3L on autophagic flux in HCC-SR cells. **A** The levels of BNIP3, BNIP3L, LC3, and β-actin in HCC-SR cells were determined using immunoblotting analysis. The LC3-II／LC3-I was labelled below the LC3 lane. **B** The expression of GFP-LC3 was determined using confocal microscopy analysis. The results suggested that overexpressing BNIP3 and BNIP3L enhanced the autophagy flux of HCC-SR cells. Scale bar: 10 µm. Cells in **A** and **B** were treated with sorafenib (10 µM) under hypoxia for 48 h after the levels of BNIP3 and BNIP3L were modulated as indicated. Full-length blots are presented in Additional File 6.

**Additional file 3**. Additional tables.

**Additional file 4**. Additional information on the methods.

**Additional file 5**. Test information and values supporting the statistical graphs.

**Additional file 6**. Full-length blots/gels.

## Data Availability

All data generated or analyzed during this study are included in this published article. RNA-seq analysis used in this study is derived from our previous work, and the methods for analysis had been explained in detail [19]. The data of RNA-seq had been submitted to the NCBI Gene Expression Omnibus (GEO; https://www.ncbi.nlm.nih.gov/geo/) under accession number GSE 120160.

## Abbreviations

RTK-MAPK: Receptor tyrosine kinase-MAP kinase
HCC: Hepatocellular carcinoma
MenSCs: Human menstrual blood-derived stem cells
SR: Sorafenib resistance
CCK-8: Cell Counting Kit -8
IC50: Half maximal inhibitory concentration
MeDIP: Methylated DNA immunoprecipitation
ATP: Adenosine triphosphate
SPF: Specific pathogen-free
ROS: Reactive oxygen species
MMP: Mitochondrial membrane potential
TEM: Transmission electron microscopy
mtDNA: Mitochondrial DNA
CQ: Chloroquine
BafA1: Bafilomycin A1
5-Aza: 5-Aza-2′-deoxycytidine
3-MA: 3-Methyladenine
BNIP3: BCL2 interacting protein 3
BNIP3L: BCL2 interacting protein 3 like
TETs: Ten-eleven translocation family
DNMTs: DNA methyltransferase family
HIF-1α: Hypoxia inducible factor 1 subunit alpha
CoxIV: Cytochrome c oxidase-IV
ATG14: Autophagy related 14
VPS34: Phosphatidylinositol 3-kinase catalytic subunit type 3
