# Supplementary figures and images for: Human menstrual blood-derived stem cells reverse sorafenib resistance in hepatocellular carcinoma cells through the hyperactivation of mitophagy
Source: Stem Cell Res Ther. 2023 Apr 1;14:58. doi: 10.1186/s13287-023-03278-8 (PMC10068152; doi:10.1186/s13287-023-03278-8)

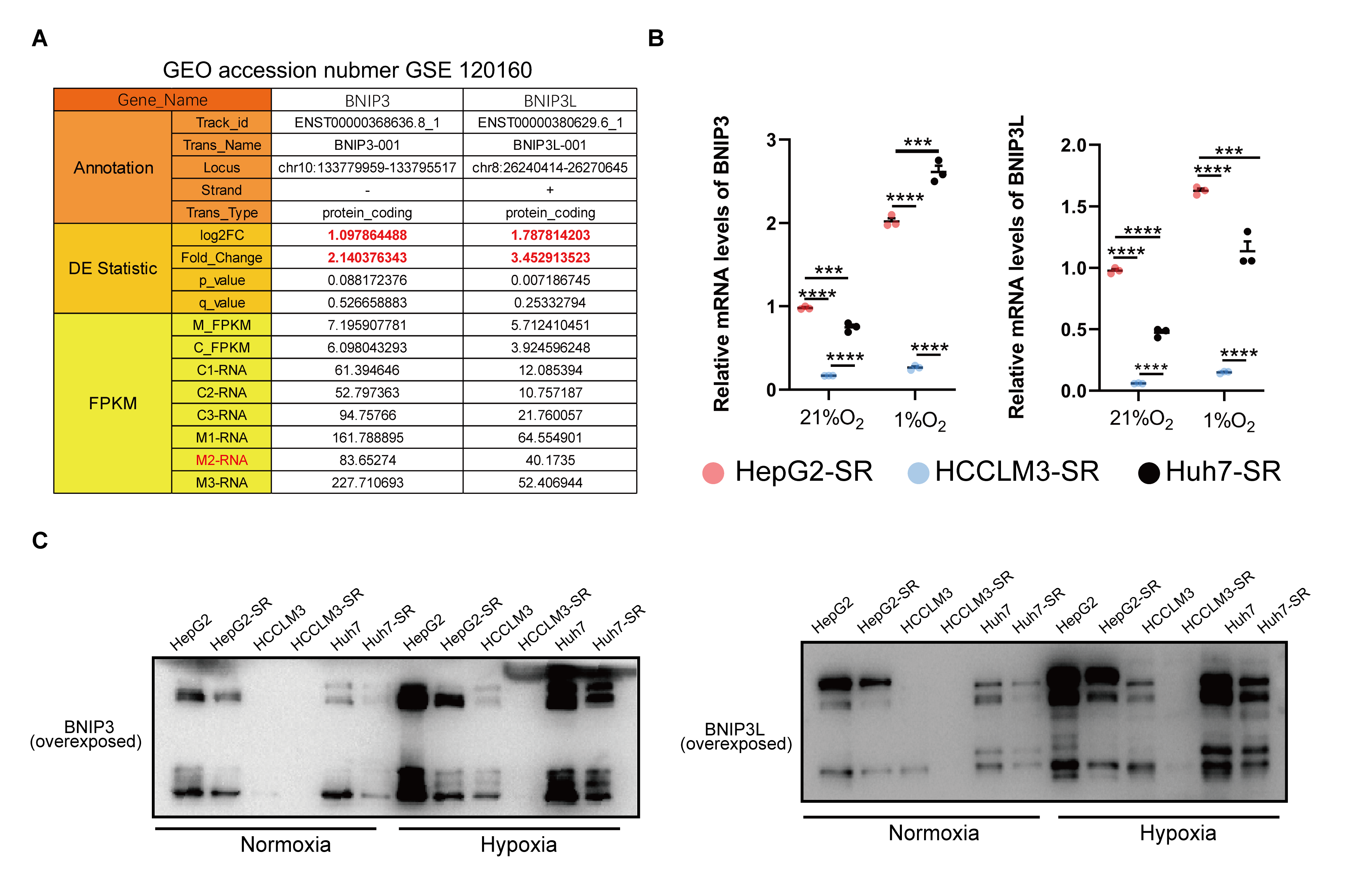

Supplement: Supplementary file 1 — Additional file 1. Figure S1. RNA-seq results and characterization of MenSCs. A RNA-seq analysis of BNIP3 and BNIP3L mRNA expression in HepG2 in response to coculture with MenSCs for 72 h. C means control group, M means MenSCs coculture group (n=3). B The mRNA levels of BNIP3 and BNIP3L were determined using qRT-PCR analysis 48 h after HCC-SR cells were cultured under normoxia or hypoxia (1% O2). C The overexposed images of the Immunoblotting analysis of BNIP3 and BNIP3L in Figure 1C. ***p < 0.001, ****p < 0.0001. [file 13287_2023_3278_MOESM1_ESM.png]

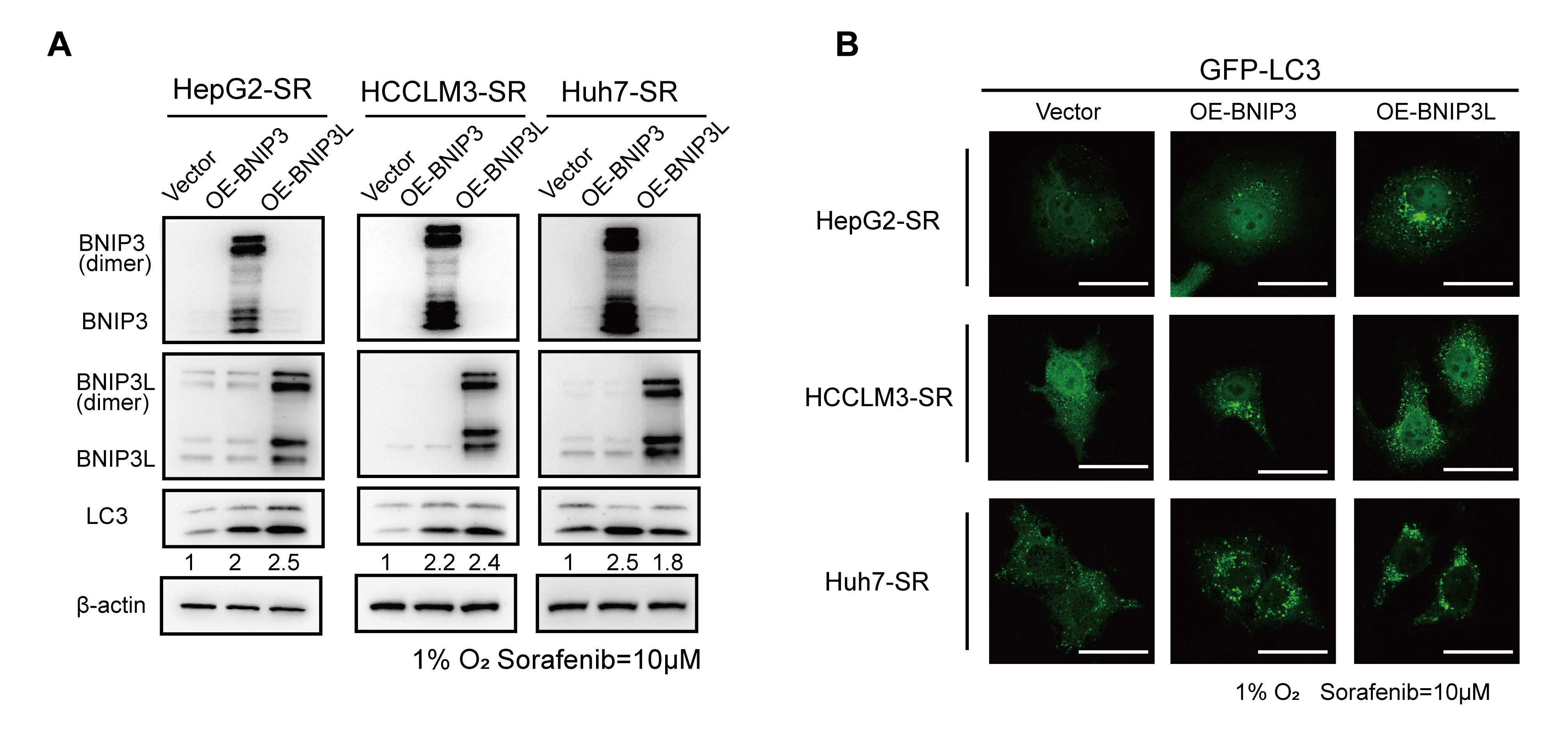

Supplement: Supplementary file 2 — Additional file 2. Figure S2. Effects of BNIP3 and BNIP3L on autophagic flux in HCC-SR cells. A The levels of BNIP3, BNIP3L, LC3, and β-actin in HCC-SR cells were determined using immunoblotting analysis. The LC3-II／LC3-I was labelled below the LC3 lane. B The expression of GFP-LC3 was determined using confocal microscopy analysis. The results suggested that overexpressing BNIP3 and BNIP3L enhanced the autophagy flux of HCC-SR cells. Scale bar: 10 µm. Cells in A and B were treated with sorafenib (10 µM) under hypoxia for 48 h after the levels of BNIP3 and BNIP3L were modulated as indicated. Full-length blots are presented in Additional File 6. [file 13287_2023_3278_MOESM2_ESM.png]
