# Supplementary material for: Human menstrual blood-derived stem cells reverse sorafenib resistance in hepatocellular carcinoma cells through the hyperactivation of mitophagy
Source: Stem Cell Res Ther. 2023 Apr 1;14:58. doi: 10.1186/s13287-023-03278-8 (PMC10068152; doi:10.1186/s13287-023-03278-8)
Supplement: Supplementary file 3 — Additional file 3. Additional tables. [file 13287_2023_3278_MOESM3_ESM.docx]

**Additional Table 1 The density of MenSCs and HCC cells for different experimental purposes.**

| **Co-culture system** | **Experimental purposes** | **The density of MenSCs per Transwell-permeable supports** | **The density of HCC cells per well** |
| --- | --- | --- | --- |
| **6-well** | **Protein extraction** | **3×10^5^** | **3×10^5^** |
|  | **Flow cytometric analyses** |  |  |
|  | **MeDIP after co-culture** |  |  |
|  | **ATP measurement** |  |  |
|  | **Transmission electron microscopy** |  |  |
|  | **Clone formation** | **2×10^4^** | **1×10^3^** |
| **12-well** | **RNA extraction** | **1×10^5^** | **1×10^5^** |
|  | **Cell viability measurement** |  |  |
|  | **Immunofluorescence** | **2×10^4^** | **2×10^4^** |

**Additional Table 2 list of primers.**

| **Quantitative and semi-quantitative analysis of *BNIP3* and *BNIP3L*** | |
| --- | --- |
| Primer name | Sequence (5’-3’) |
| BNIP3-F | GGTCAAGTCGGCCGGAAAATAT |
| BNIP3-R | CGCCTTCCAATATAGATCCCCAA |
| BNIP3L-F | ATGTCGTCCCACCTAGTCGAG |
| BNIP3L-R | TGAGGATGGTACGTGTTCCAG |
| β-actin-F | GTGGGGCGCCCCAGGCACCA |
| β-actin-R | CTTCCTTAATGTCACGCACGATTTC |
| **Quantitative analysis of methylases and demethylases** | |
| Primer name | Sequence (5’-3’) |
| DNMT1-F | GGCTGAGATGAGGCAAAAAG |
| DNMT1-R | ACCAACTCGGTACAGGATGC |
| DNMT3A-F | TATTGATGAGCGCACAAGAGAGC |
| DNMT3A-R | GGGTGTTCCAGGGTAACATTGAG |
| DNMT3B-F | AATGTGAATCCAGCCAGGAAAGGC |
| DNMT3B-R | ACTGGATTACACTCCAGGAACCGT |
| TET1-F | TCTTCCCCATGACCACATCT |
| TET1-R | GAGGGAAAAGAAGCCCAAAG |
| TET2-F | ACGCTTGGAAGCAGGAGAT |
| TET2-R | CACAAGGCTGCCCTCTAGTT |
| TET3-F | CCCACAAGGACCAGCATAAC |
| TET3-R | CCATCTTGTACAGGGGGAGA |
| β-actin-F | GTGGGGCGCCCCAGGCACCA |
| β-actin-R | CTTCCTTAATGTCACGCACGATTTC |
| **Quantitative analysis of *BNIP3* and *BNIP3L* promoter methylation levels** | |
| Primer name | Sequence (5’-3’) |
| BNIP3-F | TAGCAGGATGGAAAGACGGG |
| BNIP3-R | TCTCCTTTGAAGGGGCGGT |
| BNIP3L-F | TCCTAGATGGAGGGTGGTGG |
| BNIP3L-R | GTCATACCCGGAAGCAGGAG |

**Additional Table 3 list of shRNAs.**

| Name | sense (5’-3’) |
| --- | --- |
| shBNIP3-1 | GCCTCGGTTTCTATTTATAAT |
| shBNIP3-2 | GAACTGCACTTCAGCAATAAT |
| shBNIP3L-1 | CAGTCAGAAGAAGAAGTTGTA |
| shBNIP3L-2 | GCTAGGCATCTATATTGGAAA |
| shTET2-1 | GCGTTTATCCAGAATTAGCAA |
| shTET2-2 | CCTCAAGCATAACCCACCAAT |
| shTET2-3 | CCTTATAGTCAGACCATGAAA |
| shTET2-4 | GCCAAGTCATTATTTGACCAT |
| shNC | CAACAAGATGAAGAGCACCAA |
