# Supplementary material for: Human menstrual blood-derived stem cells reverse sorafenib resistance in hepatocellular carcinoma cells through the hyperactivation of mitophagy
Source: Stem Cell Res Ther. 2023 Apr 1;14:58. doi: 10.1186/s13287-023-03278-8 (PMC10068152; doi:10.1186/s13287-023-03278-8)
Supplement: Supplementary file 4 — Additional file 4. Additional information on the methods. [file 13287_2023_3278_MOESM4_ESM.docx]

**Additional methods**

**qRT**‒**PCR and RT**‒**PCR**

Quantitative analysis of mRNA levels was performed by Quantitative Real‒Time PCR (qRT‒PCR). Briefly, total RNA was extracted by MiniBEST Universal RNA extraction kit (TaKaRa Bio Inc., Beijing, China). cDNA was then reverse-transcribed using PrimeScript^TM^RT Master Mix (TaKaRa Bio Inc.). qRT‒PCR was performed using TB Green ^®^ *Premix* ^Ex^ Taq^TM^ (TaKaRa Bio Inc.). CFX-96 system (Bio-Rad, CA, USA) and LightCycler480 II (Roche Life Science, CH) were used to perform qRT‒PCR reactions.

Semi-quantitative analysis of *BNIP3* and *BNIP3L* transcription was performed by reverse transcription-PCR (RT‒PCR). Total RNA was reverse transcribed using PrimeScript™ RT-PCR Kit (TaKaRa Bio Inc.) and amplified by Thermocycler (Thermo Fisher Scientific). PCR products were electrophoresed on 2% agarose gel.

All primer sequences used in this study are listed in the Additional file 3, Table 2.

**Immunoblotting**

Total protein was extracted from cells using 10×RIPA Lysis Buffer (Merck Millipore, Germany) with a phosphatase and protease inhibitor cocktail (Thermo Fisher Scientific). After 30 mins of lysed on ice followed by centrifugation at 15,000 g for 15 mins at 4 ℃, the supernatant (cell lysates) was collected. The protein concentration was measured using a BCA protein concentration kit (Beyotime). Proteins were denatured using 5×loading buffer (Fude Biological Technology CO., Hangzhou, China) and boiled at 100 °C for 5 mins. Immunoblotting was performed following standard WB procedures as previously described(17). Antibodies against HIF-1α (ab51608, 1:1000) and TET1 (ab191698, 1:1000) were purchased from Abcam (Cambridge, UK); antibodies against BNIP3(#44060, 1:1000), BNIP3L (#12396, 1:1000), LC3A/B (#12741, 1:1000), Bcl2 (#4223, 1:1000), Beclin1(#3495, 1:1000), ATG14 (#96752, 1:1000), VPS34 (#4263, 1:1000), COX IV (#11967, 1:1000), and TET2 (#18950, 1:1000) were purchased from Cell Signaling Technology (Danvers, MA, USA); antibody against β-actin (1:5000) was purchased from EarthOX (San Francisco, CA, USA).

**Flow cytometric analyses**

Cell apoptosis was detected using the Annexin V/PI detection kit (BD Biosciences, CA, USA) as previously described(18). Briefly, 1×10^5^ were collected after trypsin (without EDTA) digestion and resuspended with fresh complete media. Cells were then stained with PI and Annexin V according to the manufacturer’s instructions.

Reactive oxygen species (ROS) levels were detected using a DCFH-DA-based Reactive Oxygen Species Assay Kit (Beyotime). 1×10^5^ were collected after trypsin digestion and resuspended with fresh complete media. Cells were then stained with DCFH-DA according to the manufacturer’s instructions.

All samples were analysed on a Novocyte flow cytometer (ACEA Biosciences, CA, USA).

**Plasmid transfection**

The plasmids pCMV3-BNIP3 and pCMV3-BNIP3L were purchased from Sinobiological (Beijing, China) to overexpress BNIP3 and BNIP3L. The plasmids pCMV-mCherry-GFP-LC3 and pCMV-GFP-LC3 were generously provided by Dr. Xinyi Wang (School of medicine, Zhejiang University) to perform autophagic analysis. The pLKO.1-based shRNAs corresponding to human BNIP3, BNIP3L and TET2 were purchased from Sigma-Aldrich. Details on the shRNAs are shown in the Additional file 3, Table 3. Plasmids were transfected by Lipofectamine 3000 (Thermo Fisher Scientific) according to the manufacturer’s instructions.

**Autophagic analysis**

For LC3-II degradation assays, cells were collected after treatments, proteins were collected and subject to immunoblotting. The relative greyscale was measured by ImageJ, and the ratio of LC3-II／LC3-I was then calculated.

For autophagosome maturation assays, cells were transfected with pCMV-mCherry-GFP-LC3 and collected after treatment. Since the GFP fluorescence is quenched in lysosomes caused by its lysosomal acidic pH, mCherry-positive GFP-positive puncta (mCherry^+^, GFP^+^) represent autophagosomes whereas mCherry-positive GFP-negative puncta (mCherry^+^, GFP^-^) represent autolysosomes. Autophagic flux was calculated by the ratio of (the number of red puncta – the number of yellow puncta) to the number of red puncta. The images were captured using an OLYMPUS IX83-FV3000-OSR confocal laser scanning microscope (Olympus Corporation, Tokyo, Japan) and OLYMPUS FV31S-SW acquisition software (Olympus Corporation, Tokyo, Japan). The resolution of each image was 1024×1024.

For mitophagy detection, mitochondria and lysosomes were respectively stained by MitoTracker and LysoTracker (Beyotime) and captured using an OLYMPUS IX83-DP70 fluorescence microscopy (Olympus Corporation, Tokyo, Japan) and CellSens Standard acquisition software (Olympus Corporation, Tokyo, Japan). The resolution of each image was 640×640.

**Immunohistochemistry**

Immunohistochemistry was performed using standard procedures according to the manufacturer’s instructions. Briefly, xenograft tumour sections were deparaffinized with xylene (three washes for 5 mins each) and rehydrated with an alcohol gradient (two washes with 100% ethanol for 10 mins each and two washes with 95% ethanol for 10 mins each). After washing the sections two times with dH_2_O for 5 mins each, endogenous peroxidase was then blocked with 3% H_2_O_2_ for 10 mins. Next, antigen unmasking was performed with citrate acid repair buffer (pH = 6.0) at 95 °C for 30 min. Afterwards, tumour sections were blocked with 5% normal goat serum (Beyotime) for 1 h, washed three times with PBS, and incubated overnight with one of the following primary antibodies: anti-TET2 (1:50, Abcam, ab243323), anti-Ki67 (1:200, Abcam, ab15580), anti-BNIP3L (1:100, Abcam, ab155010), anti-BNIP3 (1:100, Cell Signaling Technology, #44060), and anti-CoxIV (1:50, Cell Signaling Technology, #11967) antibodies. Sections were then incubated with horseradish peroxidase (HRP)-conjugated secondary antibodies (Thermo Fisher Scientific) at room temperature for 1 h and developed with a DAB kit (Thermo Fisher Scientific). The Immunohistochemistry images were captured using an OLYMPUS IX83-DP70 fluorescence microscopy and CellSens Standard acquisition software. The resolution of each image was 1360×1024. The average optical density of TET2, BNIP3, BNIP3L, and COX IV were analysed using ImageJ software (NIH, USA). Ki67-positive cells were manually counted. Researchers analysing the data were blinded to the group allocation until all the statistical results were finally obtained.

**Immunofluorescence**

Cells after treatments were washed with PBS and fixed with ice methanol for 7 mins at room temperature. Cells were then permeabilized with 0.3% TritonX-100 (Sigma-Aldrich) in PBS for 20 mins at room temperature followed by blocking with 10% goat serum (Beyotime) in 0.3% TritonX-100/PBS for 1 h. Cells were incubated with primary antibodies diluted in the blocking buffer at 4 ℃ overnight. On the next day, cells were washed with PBS three times and then incubated with secondary antibodies diluted in the blocking buffer at room temperature for 1 h in the dark. Cells were mounted in Prolong Gold Antifade with DAPI (Invitrogen) overnight in the dark.

Primary antibodies against BNIP3(#44060, 1:800), BNIP3L (#12396, 1:400), COX IV (#11967, 1:200) were purchased from Cell Signaling Technology. Secondary antibodies Goat anti-Mouse IgG (H+L) Cross-Adsorbed Secondary Antibody, Alexa Fluor 488 (A11029, 1:200) and Goat anti-Rabbit IgG (H+L) Cross-Adsorbed Secondary Antibody, Alexa Fluor 568 (A11036, 1:200) were purchased from Thermo Fisher Scientific.

The images were captured using an OLYMPUS IX83-FV3000-OSR confocal laser scanning microscope and OLYMPUS FV31S-SW acquisition software. The resolution of each image was 1024×1024.

**Co-immunoprecipitation (Co-IP)**

HCC-SR cells were seeded at 70% confluency in 6-well plates. Cells after indicated treatments were lysed using Co-IP lysis buffer (Pierce™ IP, Thermo Fisher Scientific) with a protease inhibitor cocktail (Thermo Fisher Scientific) followed by centrifugation at 15,000 g for 15 mins at 4 ℃. Supernatants were collected and incubated with antibodies and protein A/G magnetic beads (Pierce™ IP, Thermo Fisher Scientific) together at 4℃ for 6 h according to the manufacturer’s instructions, IgG served as a negative control. Input solution and IP solution were denatured using 5×loading buffer and boiled at 100°C for 5 mins. Immunoblotting was performed following standard WB procedures to examine the target proteins and their interacted proteins.

Antibodies: Bcl2 (Cell Signaling Technology, #15071, 1:50), Beclin1 (Cell Signaling Technology, #4122, 1:100), and TET2 (Cell Signaling Technology, #18950, 1:100).

**Transmission electron microscopy**

Cells were collected after treatment and fixed with 2.5% glutaraldehyde at 4 °C overnight. After that, cells were washed with 0.1 M PBS three times and fixed with osmic acid for 1 h. After the second fixation, cells were washed with ddH20 three times and further fixed with 2% uranyl acetate for 30 mins. After serial dehydration in a series of ethanol gradients (50%-100%) and acetone (100%), the subsequent steps of sample preparation were entrusted to the Center of Cryo-Electron Microscopy (Zhejiang University, Hangzhou, China). Micrographs were captured using a Tecnai G2 spirit 120kV transmission electron microscope (Thermo FEI, Eindhoven, NL) and Gatan DigitalMicrograph (Gatan, Inc., PA, USA) acquisition software. The resolution of each image was 2672×2672.

**Methylated DNA Immunoprecipitation (MeDIP)**

MeDIP was performed with a Methylated DNA Immunoprecipitation ChIP Kit (Abcam, ab117135) according to the manufacturer's instructions. Briefly, cells after treatments were lysed by Lysis Buffer and incubated for 5 min at room temperature. Genomic DNA in the cell solution was then sheared by sonication followed by centrifugation at 14,000 rpm for 10 min. The DNA fragments were immunoprecipitated with an anti-5-methylcytosine antibody. IgG served as a negative control. After a series of isolation and purification, the methylated DNA was collected by elution with a 1 X TE Buffer (PH 8.0).

For MeDIP-qPCR, 10% of input DNA was used as control. The IP efficiency was calculated as the per cent of input DNA immunoprecipitated (% input), which used the Ct values obtained for the target promoter region from input DNA and mDNA (methylated DNA). Equation for %input in this work: % Input = 10%×2 ^[Ct(input) - Ct(mDNA)]^. The results were further normalized to the parental cell lines (Figure 1E), and the NC group (Figure 5A). Primers were listed in Additional file 3, Table 2.

**Total cellular ATP content, mitochondrial membrane potential**

Total cellular ATP content and mitochondrial membrane potential (MMP) levels were respectively detected by an Enhanced ATP Assay Kit (Beyotime) and Enhanced mitochondrial membrane potential assay kit with JC-1 (Beyotime) according to the manufacturer's instructions. The JC-1 images were captured using an OLYMPUS IX83-DP70 fluorescence microscopy and CellSens Standard acquisition software. The resolution of each image was 1360×1024.
