# Supplementary material for: Human menstrual blood-derived stem cells reverse sorafenib resistance in hepatocellular carcinoma cells through the hyperactivation of mitophagy
Source: Stem Cell Res Ther. 2023 Apr 1;14:58. doi: 10.1186/s13287-023-03278-8 (PMC10068152; doi:10.1186/s13287-023-03278-8)

Figures 1C, S1C

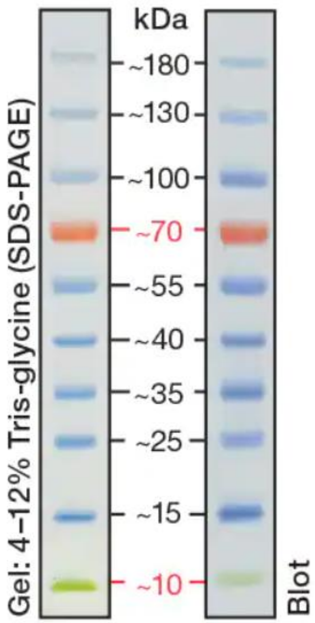


Thermo 26616

| Protein | MW(kDa) |
|---|---|
| BNIP3 | 22-28,50-55 |
| BNIP3L | 38,76 |
| HIF-1α | 120 |
| β-actin | 43 |

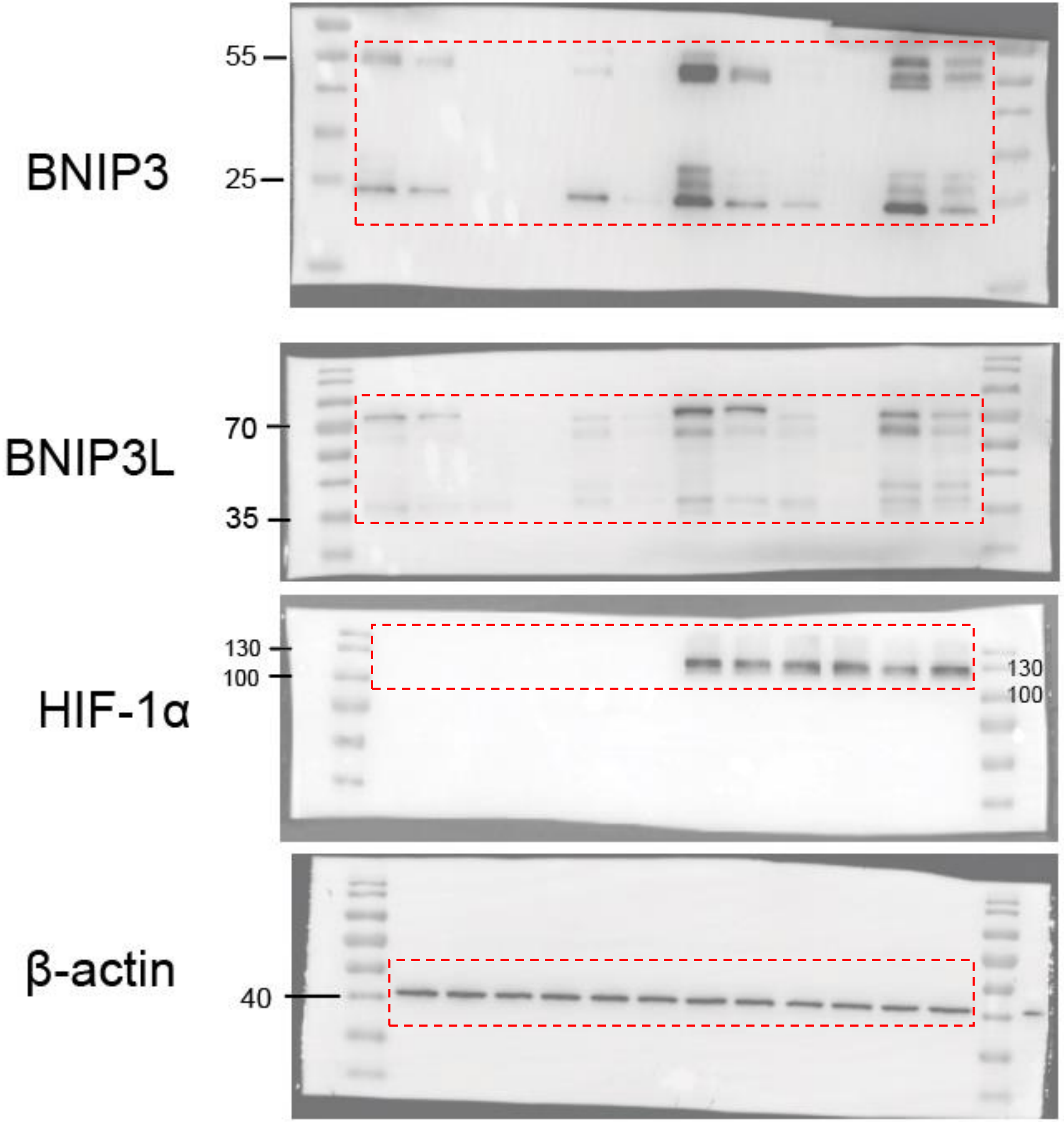

Figure 1D

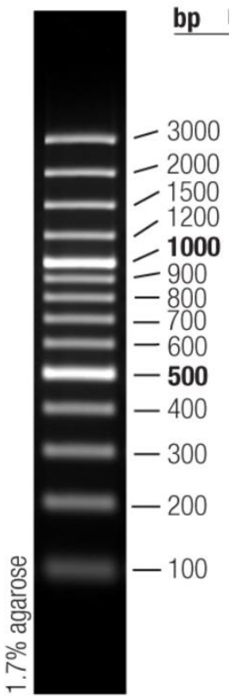


Thermo SM0321

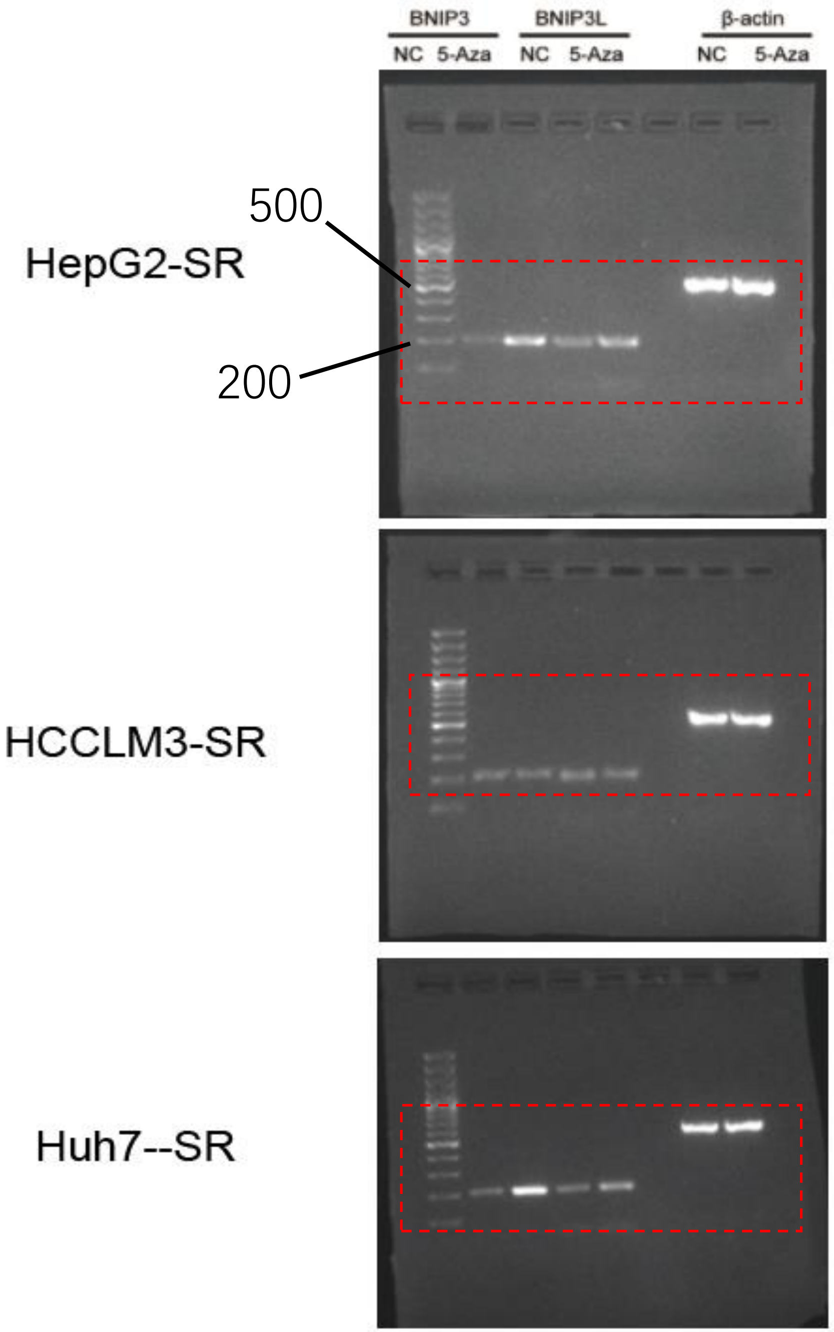


| Transcript | Product length(bp) |
|---|---|
| BNIP3 | 199 |
| BNIP3L | 189 |
| β-actin | 541 |

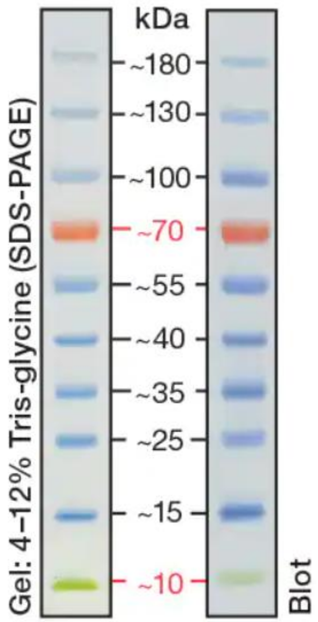


Thermo 26616

Figure 3A

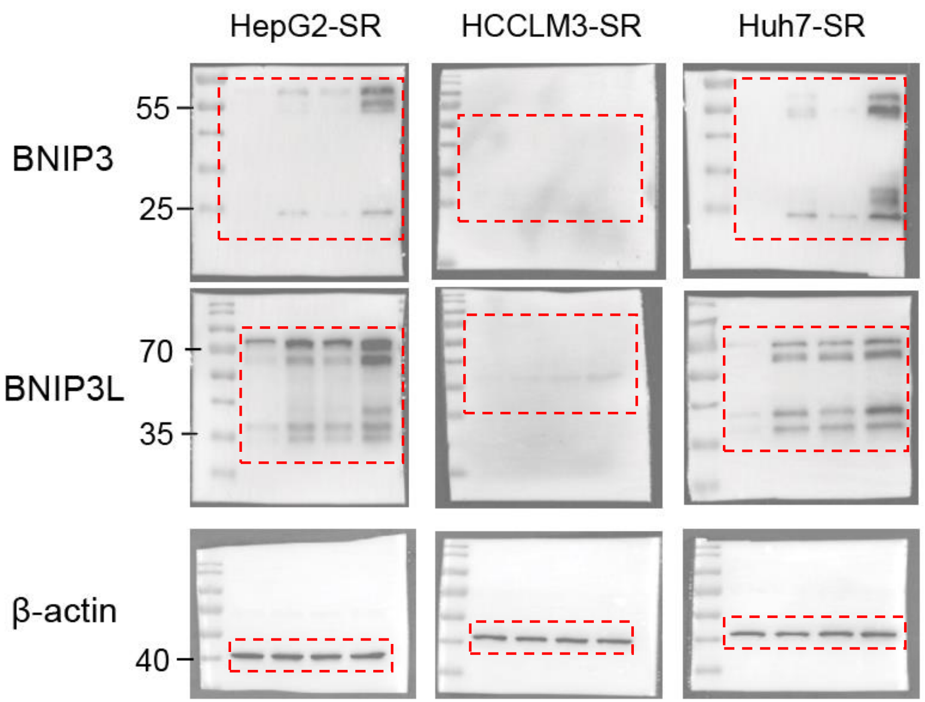


Figure 3G

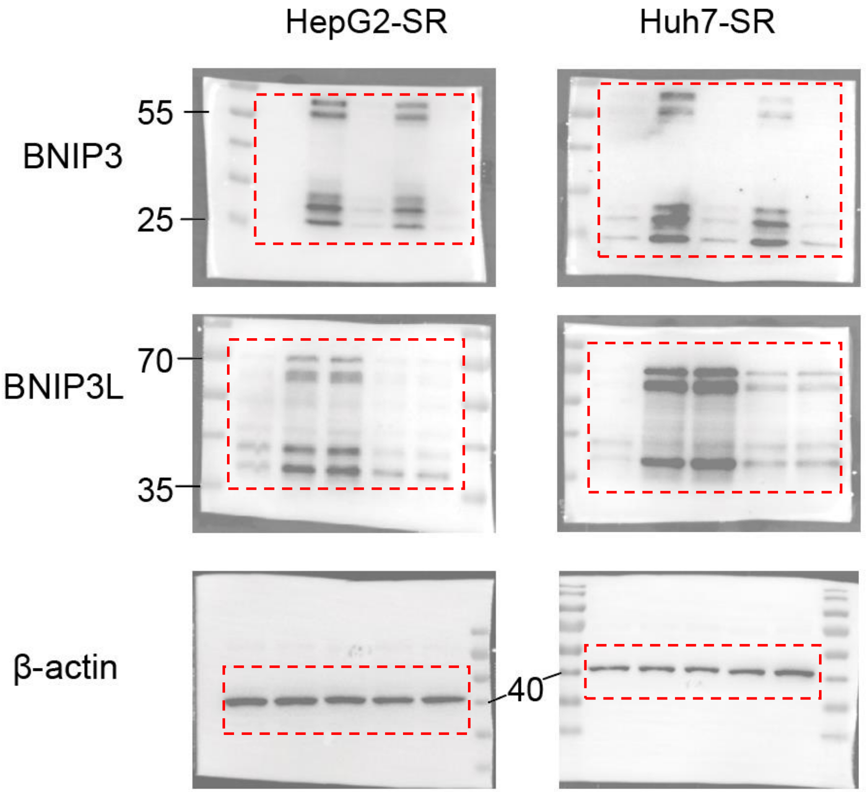


| Protein | MW(kDa) |
|---|---|
| BNIP3 | 22-28,50-55 |
| BNIP3L | 38,76 |
| β-actin | 43 |

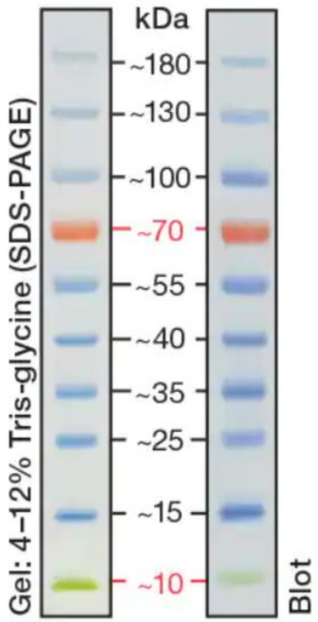


Thermo 26616

Figure 4A

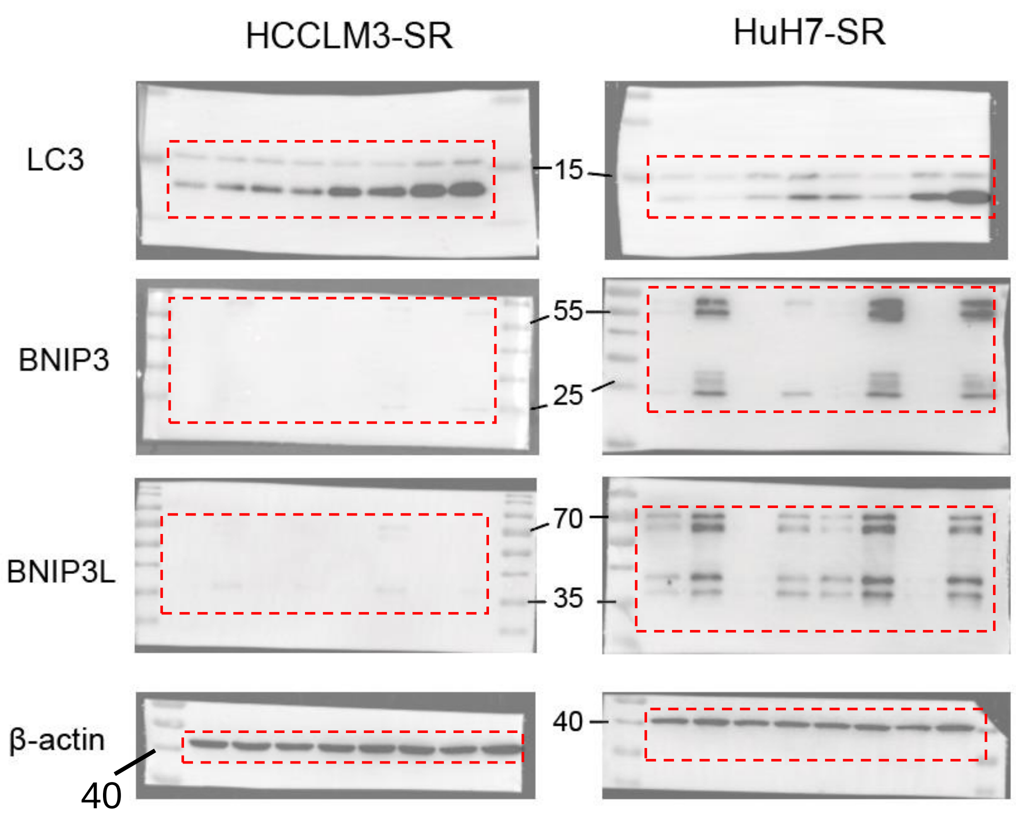


Figure 4D

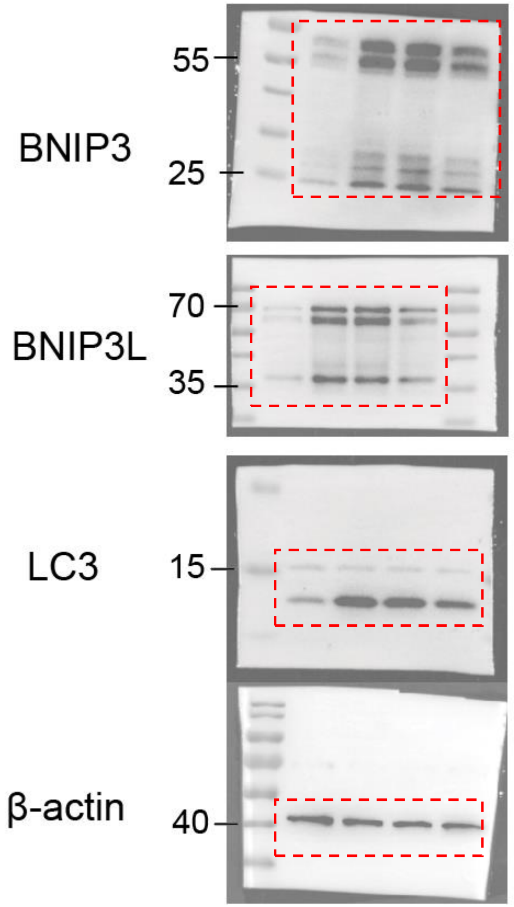


| Protein | MW(kDa) |
|---|---|
| BNIP3 | 22-28,50-55 |
| BNIP3L | 38,76 |
| β-actin | 43 |
| LC3 | 14,16 |

## Figure 4G

## Figure 4H

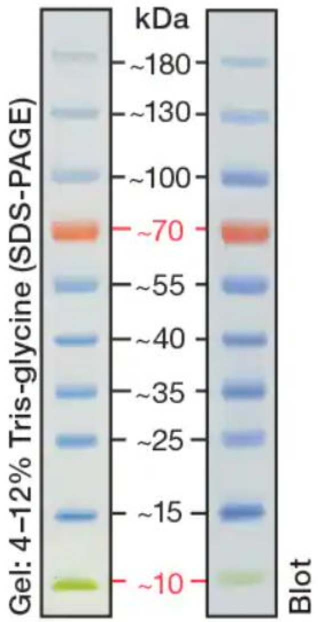


Thermo 26616

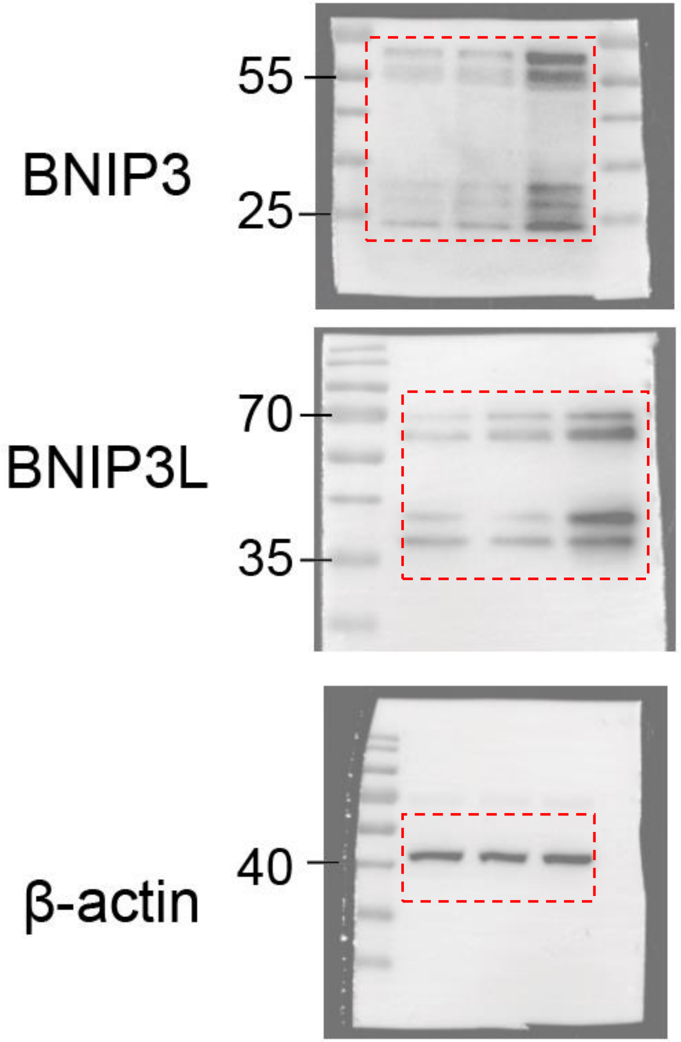


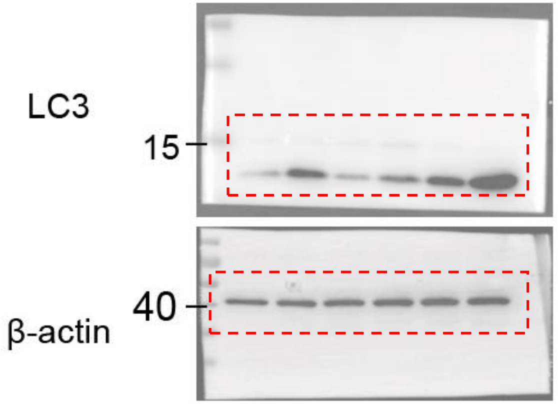


| Protein | MW(kDa) |
| --- | --- |
| BNIP3 | 22-28,50-55 |
| BNIP3L | 38,76 |
| β-actin | 43 |
| LC3 | 14,16 |

Figure 4J

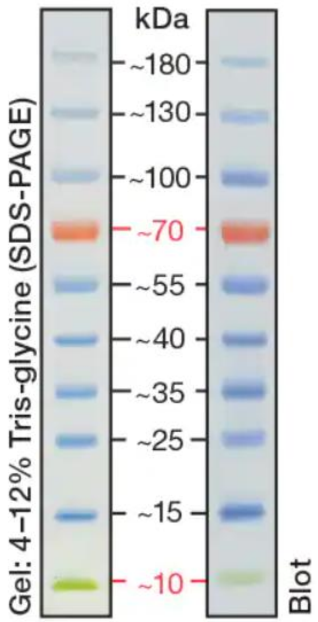


Thermo 26616

| Protein | MW(kDa) |
|---|---|
| BNIP3 | 22-28, 50-55(dimer) |
| BNIP3L | 38,76(dimer) |
| Beclin1 | 60 |
| BCL2 | 26 |
| ATG14 | 65 |
| VPS34 | 100 |

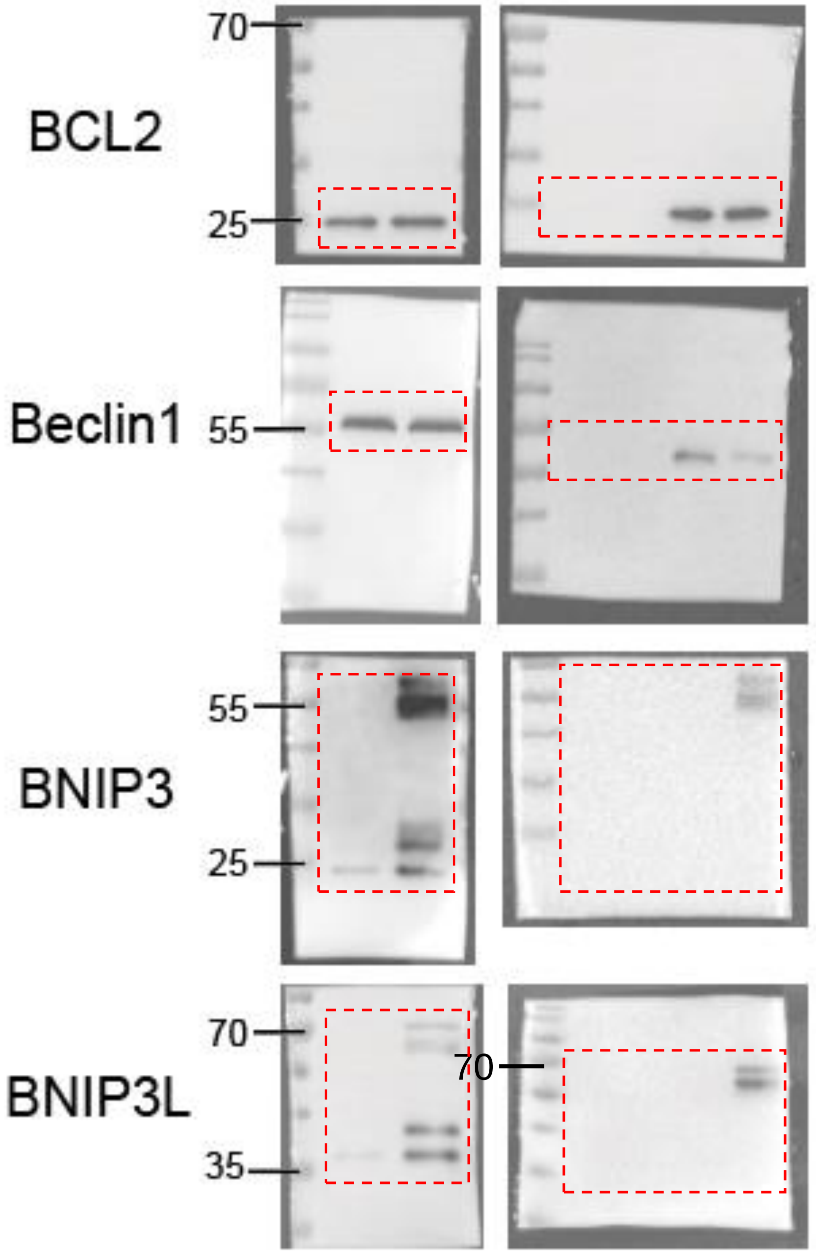


Figure 4K

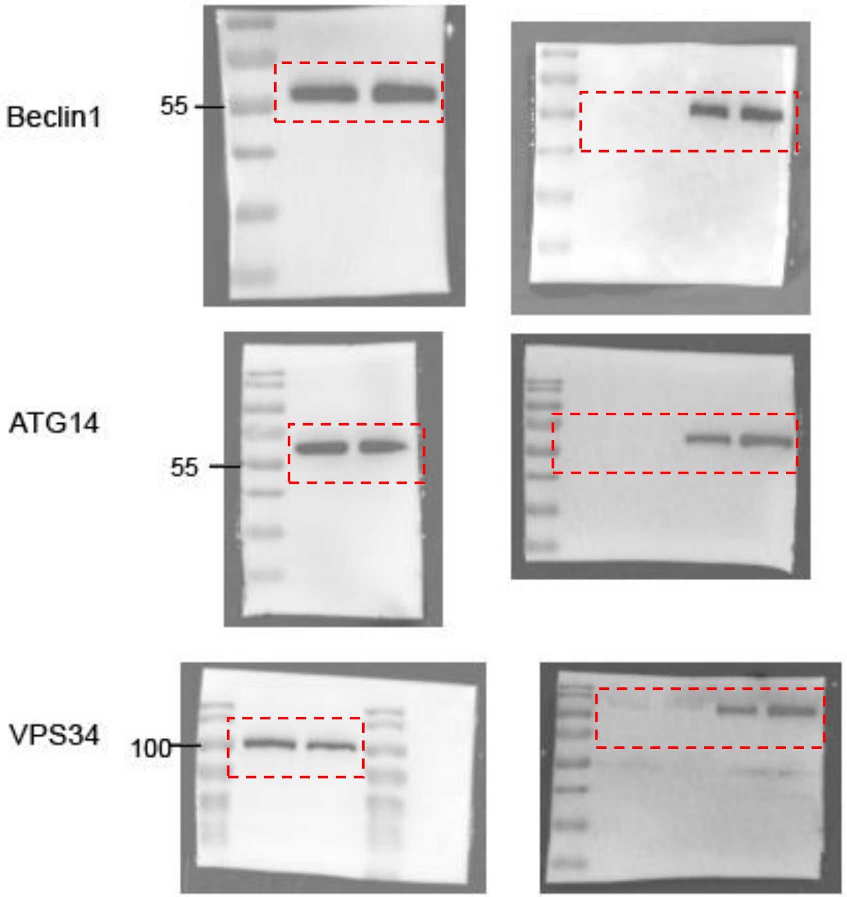

# Figure 5C

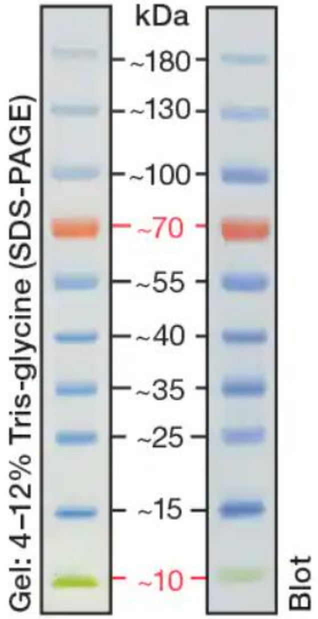


Thermo 26616

| Protein | MW(kDa) |
|---|---|
| BNIP3 | 22-28,50-55 |
| BNIP3L | 38,76 |
| CoxIV | 17 |
| β-actin | 43 |

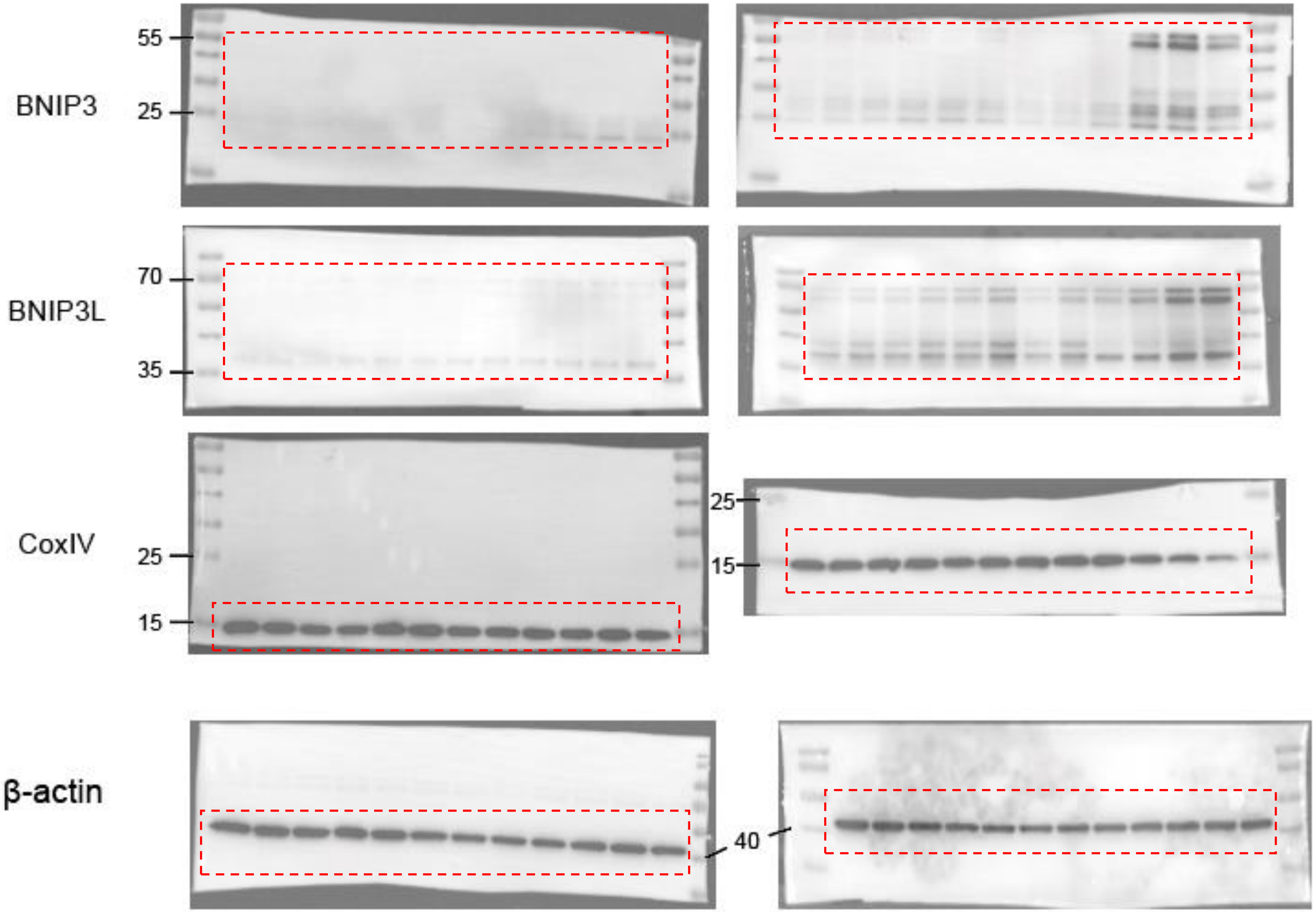

# Figure 6C

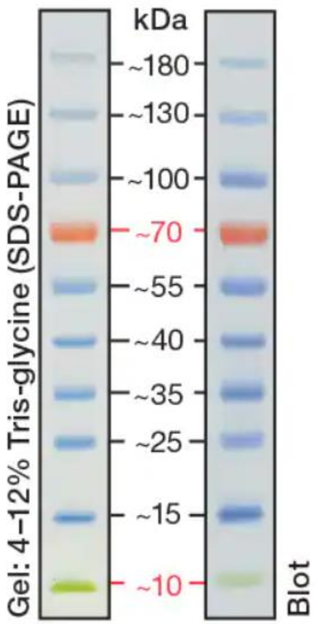


Thermo 26616

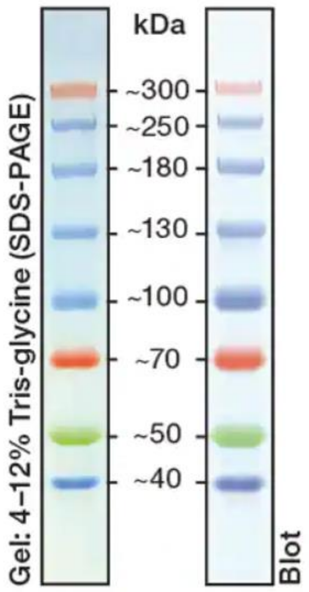


Thermo 26625

| Protein | MW(kDa) |
|---|---|
| DNMT1 | 200 |
| TET1 | 235-300 |
| TET2 | 280 |
| β-actin | 43 |

DNMT1, TET1, TET2: Thermo 26625

β-actin: Thermo 26616

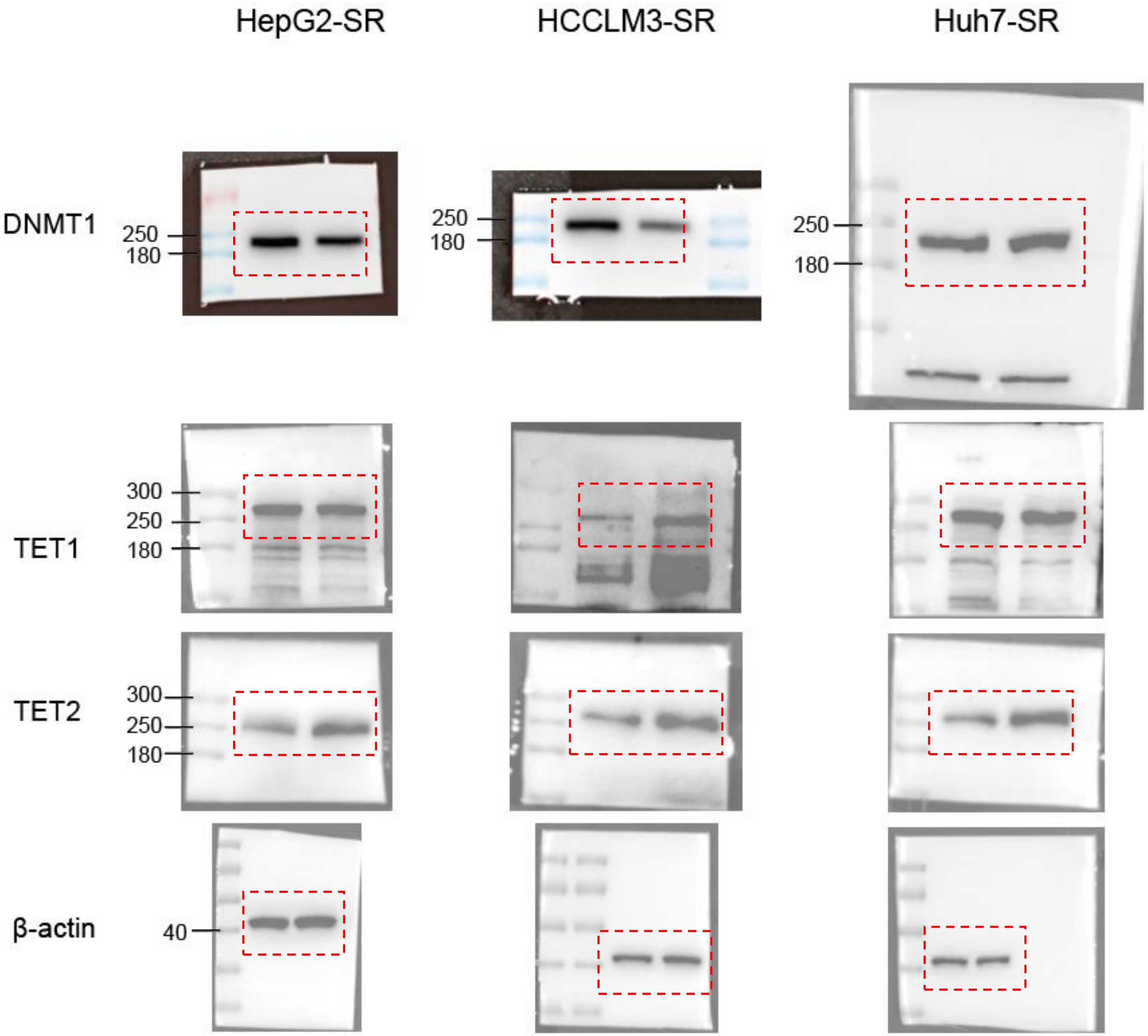

Figure 6E

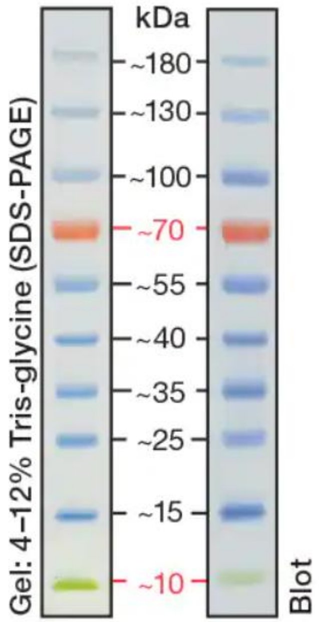


Thermo 26616

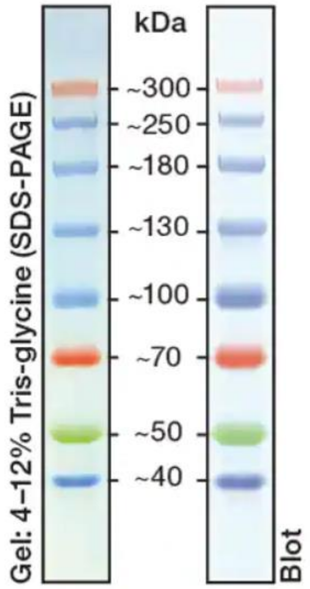


Thermo 26625

| Protein | MW(kDa) |
|---|---|
| TET2 | 280 |
| BNIP3 | 22-28,50-55 |
| BNIP3L | 38,76 |
| β-actin | 43 |

TET2 → Thermo 26625

BNIP3, BNIP3L, β-actin → Thermo 26616

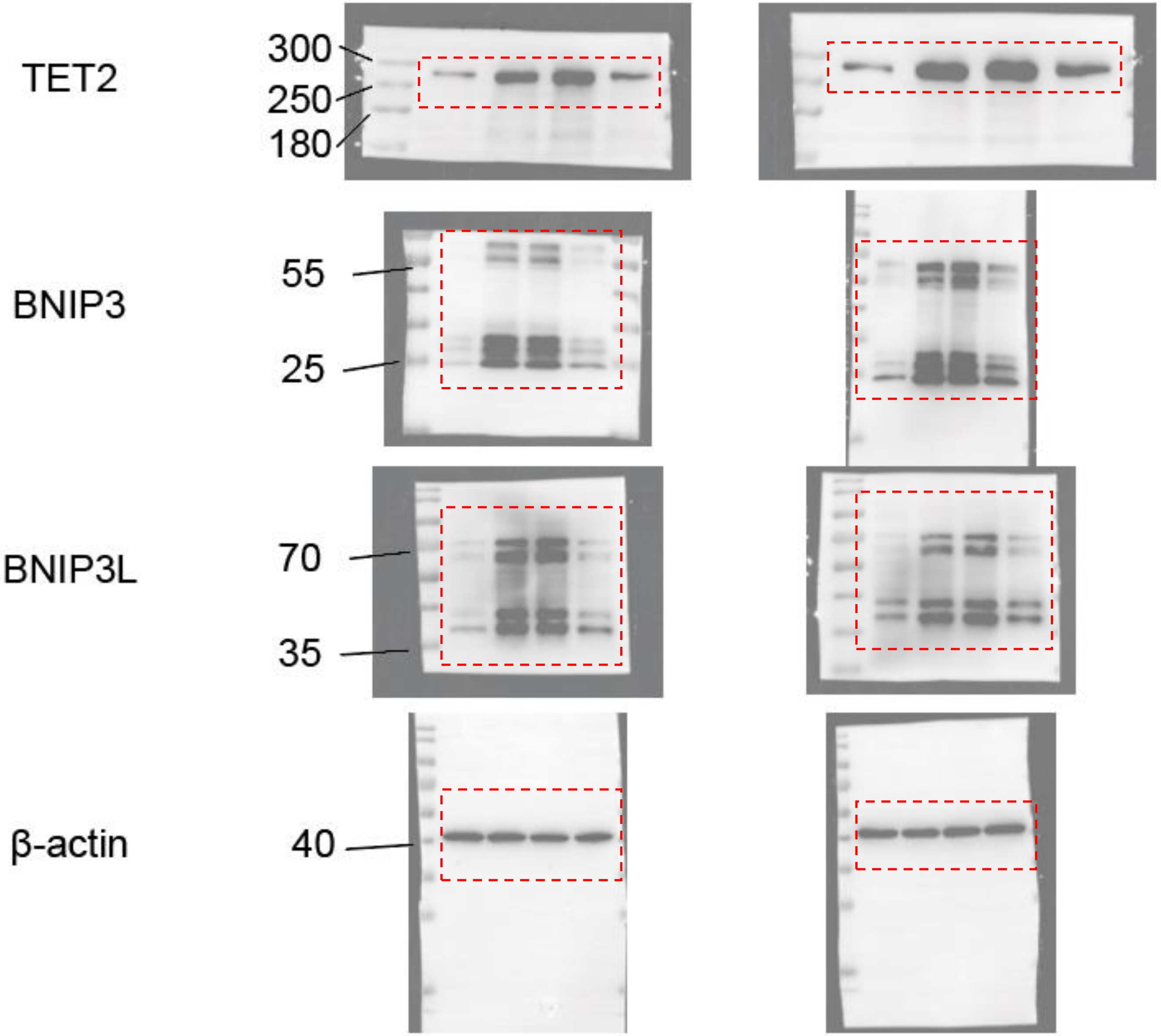

# Figure S2A

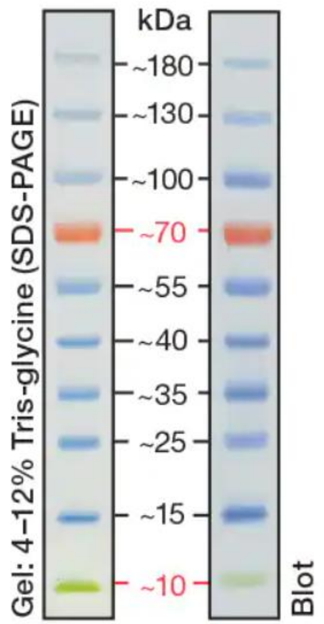


Thermo 26616

| Protein | MW(kDa) |
|---|---|
| BNIP3 | 22-28,50-55 |
| BNIP3L | 38,76 |
| β-actin | 43 |
| LC3 | 14,16 |

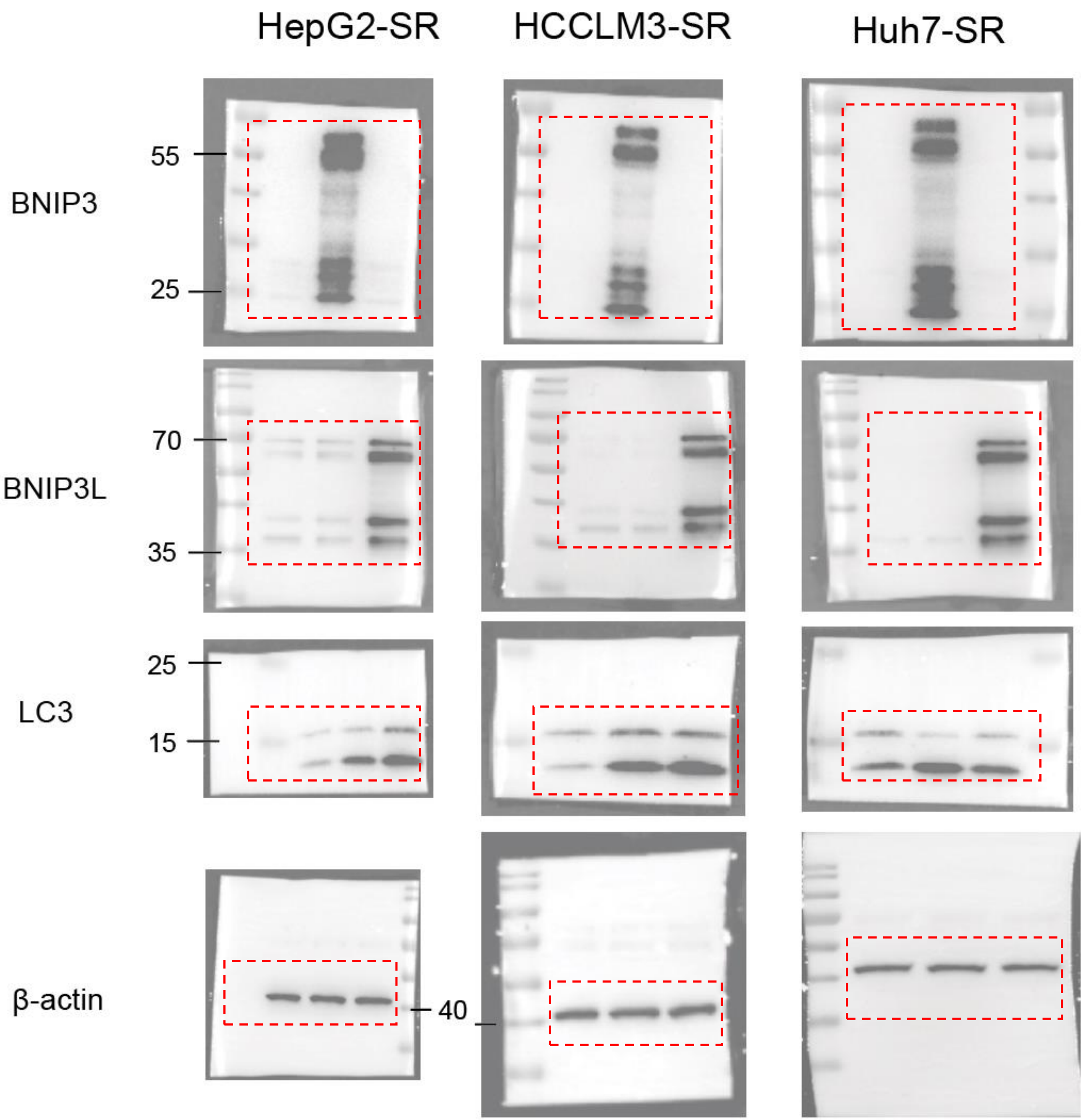

Supplement: Supplementary file 6 — Additional file 6. Full-length blots/gels. [file 13287_2023_3278_MOESM6_ESM.pdf]
